# Plant growth-promoting rhizobacterium *Bacillus megaterium* modulates the expression of antioxidant-related and drought-responsive genes to protect rice (*Oryza sativa* L.) from drought

**DOI:** 10.3389/fmicb.2024.1430546

**Published:** 2024-08-21

**Authors:** Sanghun Lee, Jung-Ae Kim, Jeongsup Song, Seonbong Choe, Geupil Jang, Yangseon Kim

**Affiliations:** ^1^Department of Research and Development, Center for Industrialization of Agricultural and Livestock Microorganisms, Jeongeup-si, Republic of Korea; ^2^School of Biological Sciences and Technology, Chonnam National University, Gwangju, Republic of Korea

**Keywords:** *Bacillus megaterium*, drought, plant growth-promoting rhizobacteria, rice, stress-responsive gene

## Abstract

Global climate change poses a significant threat to plant growth and crop yield and is exacerbated by environmental factors, such as drought, salinity, greenhouse gasses, and extreme temperatures. Plant growth-promoting rhizobacteria (PGPR) help plants withstand drought. However, the mechanisms underlying PGPR–plant interactions remain unclear. Thus, this study aimed to isolate PGPR, *Bacillus megaterium* strains CACC109 and CACC119, from a ginseng field and investigate the mechanisms underlying PGPR-stimulated tolerance to drought stress by evaluating their plant growth-promoting activities and effects on rice growth and stress tolerance through *in vitro* assays, pot experiments, and physiological and molecular analyses. Compared with *B. megaterium* type strain ATCC14581, CACC109 and CACC119 exhibited higher survival rates under osmotic stress, indicating their potential to enhance drought tolerance. Additionally, CACC109 and CACC119 strains exhibited various plant growth-promoting activities, including phosphate solubilization, nitrogen fixation, indole-3-acetic acid production, siderophore secretion, 1-aminocyclopropane-1-carboxylate deaminase activity, and exopolysaccharide production. After inoculation, CACC109 and CACC119 significantly improved the seed germination of rice (*Oryza sativa* L.) under osmotic stress and promoted root growth under stressed and non-stressed conditions. They also facilitated plant growth in pot experiments, as evidenced by increased shoot and root lengths, weights, and leaf widths. Furthermore, CACC109 and CACC119 improved plant physiological characteristics, such as chlorophyll levels, and production of osmolytes, such as proline. In particular, CACC109- and CACC119-treated rice plants showed better drought tolerance, as evidenced by their higher survival rates, greater chlorophyll contents, and lower water loss rates, compared with mock-treated rice plants. Application of CACC109 and CACC119 upregulated the expression of antioxidant-related genes (e.g., *OsCAT*, *OsPOD*, *OsAPX*, and *OsSOD*) and drought-responsive genes (e.g., *OsWRKY47, OsZIP23, OsDREB2, OsNAC066, OsAREB1,* and *OsAREB2*). In conclusion, CACC109 and CACC119 are promising biostimulants for enhancing plant growth and conferring resistance to abiotic stresses in crop production. Future studies should conduct field trials to validate these findings under real agricultural conditions, optimize inoculation methods for practical use, and further investigate the biochemical and physiological responses underlying the observed benefits.

## 1 Introduction

Global population growth and extreme climate change pose important agricultural challenges that may cause insufficient food supply worldwide (FAO, 2017). Rice (*Oryza sativa* L.), a crucial staple feeding nearly half of the global population, is grown on around 163 million hectares of land worldwide (Fukagawa and Ziska, 2019; Shahbandeh, 2024). Despite its importance, rice productivity is affected by abiotic and biotic stresses. Drought and high salinity are abiotic stresses that severely affect rice growth, development, and production. Specifically, drought is considered the most important constraint to rice production, affecting approximately 19–23 million hectares of rice fields (Kumar, 2017).

Under drought, plants exhibit changes in various properties, including physiological (e.g., photosynthesis, osmotic balance, transpiration, leaf water content, and stomatal water transmission activity), morphological (e.g., leaf area, leaf number, root length, leaf aging, maturation, and growth stage), and biochemical (e.g., antioxidant content, chlorophyll content, proline accumulation, hormonal content, and secondary metabolites) (Anjum et al., 2011; Sharma et al., 2012; Haworth et al., 2013; Ammar et al., 2015; Conesa et al., 2016; Tiwari et al., 2021; Yang et al., 2021). Although these mechanisms enable plants to minimize water loss and usage, they can adversely affect plant growth and development, consequently reducing yield (Nakabayashi and Saito, 2015).

With the urgent need to support crop growth to meet food demands amid limited water resources, interest in enhancing drought tolerance and discovering sustainable solutions to related food security challenges has increased. The acquisition of essential nutrients under drought is a significant challenge for plants, often limiting their growth and productivity. Plant growth-promoting rhizobacteria (PGPR) represent a wide variety of beneficial soil bacteria that colonize the rhizosphere and plant roots, and can grow in, on, or around plant tissues. PGPR can increase nutrient availability and uptake, directly and indirectly supporting plant growth and resilience under drought (Backer et al., 2018). Under drought, PGPR play pivotal roles in nutrient solubilization and uptake. These bacteria solubilize phosphorus and other essential nutrients, making them more accessible to plants, while also improving water content and potential, thereby enhancing mineral quality and grain yield (Creus et al., 2004; Richardson and Simpson, 2011; Hardoim et al., 2015). Nitrogen fixation by PGPR contributes to soil nitrogen enrichment, providing an additional nitrogen source for plants. This process is essential for nitrogen acquisition in nutrient-poor, dry soils (Hardoim et al., 2015). Siderophore production by PGPR is another vital mechanism that enhances nutrient acquisition under drought. Siderophores are iron-chelating compounds that microbes secrete to sequester iron from the soil environment, making it more accessible to plants. This process is especially critical under drought, where nutrient availability is limited (Sharma and Johri, 2003; Ferreira et al., 2019). In addition, PGPR can improve drought tolerance by stimulating the production of drought-resistant compounds, such as bacterial exopolysaccharides (EPS), 1-aminocyclopropane-1-carboxylate (ACC) deaminase, volatile organic compounds (VOCs), and phytohormones, such as abscisic acid (ABA), ethylene (ET), and indole-3-acetic acid (IAA) (Vejan et al., 2016; Vurukonda et al., 2016; Tiepo et al., 2018). The production of EPS by PGPR helps retain soil moisture and stabilize soil structure, further improving the water holding capacity and nutrient availability in the soil, thereby enhancing plant growth and alleviating drought (Vurukonda et al., 2016). ACC deaminase is a PGPR-produced enzyme that can modulate plant ET levels. ET is a phytohormone associated with stress responses, and high levels of ET can inhibit plant growth. ACC deaminase lowers ET levels by breaking down its precursor, ACC, thereby mitigating stress effects and promoting root elongation and nutrient uptake under drought conditions (Glick et al., 1998; Mayak et al., 2004; Vurukonda et al., 2016). VOCs emitted by soil microorganisms also enhance the ability of plants to cope with abiotic stresses, such as drought, by inducing system resistance. VOCs such as acetoin and 2,3-butanediol improve plant growth and drought resistance by modulating stress-related pathways and enhancing water use efficiency (Ryu et al., 2003; Vejan et al., 2016). Phytohormones, including ABA and ET, are critical in plant response to drought. ABA plays central roles in regulating stomatal closure, reducing water loss, and activating stress-responsive genes. Microbial interactions can influence ABA levels, enhancing the drought response of plant. While ET is involved in the regulation of growth and stress responses, its overproduction can be detrimental. Microbial modulation of ET levels via ACC deaminase activity helps maintain a balance, promoting plant growth and stress tolerance (Glick et al., 1998; Cutler et al., 2010; Cohen et al., 2015; Vurukonda et al., 2016). PGPR produce plant growth-promoting (PGP) hormones such as auxins that enhance root growth and nutrient acquisition. The best-known auxin, IAA, modifies the plant root architecture by increasing root length, root branching, and total root surface area resulting in improved water and nutrient uptake and enhanced cellular defense against water stress (Glick, 2012; Vurukonda et al., 2016). These studies indicate the promising potential of PGPR in enhancing drought tolerance.

Plants initiate specific responses that reprogram genetic, molecular, and physiological processes to mitigate the adverse effects of drought on their growth and productivity. Many plants achieve stress resistance through PGPR-mediated activation of various stress-responsive genes. Plants inoculated with PGPR under drought stress exhibit distinct gene expression profiles compared with non-inoculated plants (Saakre et al., 2017; Kaushal, 2019). For example, inoculation of sugarcane (*Saccharum* spp.) with *Gluconacetobacter diazotrophicus* PAL5 under drought activates ABA-dependent signaling genes, enhancing drought tolerance (Vargas et al., 2014). Colonization of plants by PGPR strains can markedly alter gene expression in response to drought. In rice, treatment with *Pseudomonas fluorescens* (Pf1) activates genes such as *Hsp20*, *bZIP1*, and *COC1* involved in the ABA signaling pathway, *PKDP* encoding a protein kinase, and *AP2-EREBP* associated with developmental and stress defense pathways (Saakre et al., 2017). Similarly, inoculation of *Arabidopsis thaliana* with GAP-P45 alters the expression patterns of genes related to polyamine biosynthesis (*ADC*, *AIH*, *CPA*, *SPDS*, *SPMS*, and *SAMDC*), leading to increased polyamine levels and reduced osmotic stress during drought (Sen et al., 2018). In wheat, PGPR strains such as *Arthrobacter protophormiae* SA3, *Bacillus subtilis* LDR2, and *Dietzia natronolimnaea* STR1 enhance drought and salinity tolerance by modifying the expression of genes and transcription factors related to the ET signaling pathway (Barnawal et al., 2019). In soybean, inoculation with *Pseudomonas simiae* AU under drought conditions results in the upregulation of genes encoding transcription factors (DREB/EREB), osmoprotectants (P5CS, GOLS), and water transporters (PIP, TIP), leading to improved drought tolerance compared with control plants (Vaishnav and Choudhary, 2019). Li et al. (2020) found that treating drought-stressed wheat with a cell filtrate from *Streptomyces pactum* Act12 improves growth by upregulating genes related to water deficit resistance, including those involved in cell wall expansion (*EXPA2* and *EXPA6*), proline accumulation (*P5CS*), and stomatal closure (*SnRK*) (Li et al., 2020). Additionally, application of *Bacillus subtilis* GOT9 to *A. thaliana* and *Brassica campestris* enhances drought tolerance and survival by hyper-inducing various genes responsible for improved drought tolerance phenotypes (Woo et al., 2020). Inoculation with *Pseudomonas putida* (RA) in chickpea plants under drought confers drought tolerance by differentially expressing genes involved in transcription activation (*DREB1A* and *NAC1*), stress response (*LEA* and *DHN*), reactive oxygen species (ROS) scavenging (*CAT*, *APX*, and *GST*), ET biosynthesis (*ACO* and *ACS*), salicylic acid (*PR1*), and jasmonate (*MYC2*) signaling (Tiwari et al., 2021). Similarly, potted rice plants inoculated with *Bacillus endophyticus* PB3, *Bacillus altitudinis* PB46, and *Bacillus megaterium* PB50 exhibit significant drought tolerance, particularly with strain PB50, by upregulating genes such as *LEA*, *SNAC1*, *HSP70*, *RAB16B*, and *bZIP23*, along with increased osmolyte accumulation (Devarajan et al., 2021). Recently, Omara et al. (2022) have demonstrated that inoculating rice plants with *B. megaterium*, *Pseudomonas azotoformans*, and *Rhizobium* sp. enhances growth and drought tolerance by altering the expression of genes related to growth and stress response (*COX1*, *AP2*-*EREBP*, *GRAM*, *NRAMP6*, *NAM*, *GST*, and *DHN*) and expansion (*EXP1*, *EXP2*, and *EXP3*) (Omara et al., 2022).

Bacteria, similar to plants, are also affected by hydric stress during water scarcity. Therefore, identifying and selecting drought-tolerant bacteria is valuable for developing new biostimulants suitable for water-deficient regions (García et al., 2017). *Panax ginseng* is a perennial herb of significant medicinal importance in Korea (Kim, 2018). Ginseng stands out among crops due to the high commercial value of its roots, and maintaining healthy root development is essential throughout its 4–6-year cultivation period (Durairaj et al., 2018; Ding et al., 2022). However, ginseng cultivation is frequently challenged by biotic factors, such as microbial pathogens and pests, and abiotic factors, including drought, salinity, and temperature (Foyer et al., 2016), leading to changes in microbial communities. Notably, a close relationship exists between changes in microbial communities and edaphic factors in soils where ginseng is cultivated (Punja et al., 2008; Li et al., 2018). Ginseng roots and the surrounding soil harbor a diverse microbial community, including PGPR (Ji et al., 2021). However, the specific mechanisms underlying PGPR–plant interactions and their optimization for sustainable agricultural practices remain unclear.

Thus, this study aimed to investigate the effects of bacterial strains isolated from the rhizosphere and soil of ginseng plants growing in a drought-stressed field on the growth and drought resistance of rice and elucidate the underlying mechanisms. Two bacterial strains, *Bacillus megaterium* CACC109 and CACC119, were identified for their osmotic stress tolerance and PGP traits. Various parameters, including seed germination, shoot and root growth, physiological properties, osmolyte production, expression of antioxidant-related genes and drought-responsive genes (DRGs), and soil composition, were compared between rice treated with these strains and control plants. We hypothesized that rice plants inoculated with bacterial strains isolated from the rhizosphere under drought would exhibit enhanced growth and improved drought resistance due to the beneficial impact of the bacteria on plant metabolism.

## 2 Materials and methods

### 2.1 Isolation and characterization of rhizosphere bacteria

Ginseng roots and soil were collected from a drought-stressed ginseng field (36°8′15.1″N, 127°28′3.0″E) in South Korea. The roots were washed in sterile distilled water to remove bulk soil and then transferred to new sterilized bottles containing 100 mL of 2-(*N*-morpholino)ethanesulfonic acid (MES) buffer (pH 5.7). The mixture was shaken at 200 rpm for 30 min at 27°C. Subsequently, the roots were carefully removed, and the shaken solution was stored as the rhizosphere sample. The rhizosphere solution was then centrifuged at 500 rpm for 5 min at 27°C, and the resulting supernatant was transferred to a new 50 mL conical tube and centrifuged at 8000 rpm for 15 min. The supernatant was discarded, and the resulting pellet was resuspended in 30 mL of sterile water. Bacteria were isolated using the agar plate method in combination with serial dilutions. Rhizosphere samples were diluted with sterile distilled water at a ratio of 1:10, and each dilution (0.1 mL) was cultured on Luria–Bertani (LB) broth at 30°C for 2 days. Each colony was selected and streaked onto LB agar for purification (Choi et al., 2020). The various bacterial isolates obtained from the samples were designated as “CACC” (Cialm Agricultural Cultures Collection), followed by their respective isolation numbers. The isolated bacterial cells were then grown in LB medium and stored at −80°C.

The isolated bacteria were identified based on 16S rRNA gene sequences. They were cultured in LB broth at 30°C in a shaker at 200 rpm, and the cells were collected by centrifugation at 5,000 rpm for 10 min and 4°C. Genomic DNA was extracted using the ExiPrep™ Plus Bacteria Genomic DNA Kit (BIONEER, Daejeon, South Korea) following the manufacturer’s protocols. The 16S rRNA gene was amplified from the genomic DNA using the universal primers 518F (CCAGCAGCCGCGGTAATAC) and 805R (GACTACCAGGGTATCTAATC), followed by a BLAST search in the National Center for Biotechnology Information (NCBI) database to identify sequence homologies.

#### 2.1.1 Phylogenetic analysis of *Bacillus* spp.

The full-length 16S rRNA gene sequences from the bacterial strains were compared with the sequences available in the NCBI database.^1^ The sequences were aligned using Multiple Sequence Comparison by Log-Expectation (Edgar, 2004). A phylogenetic tree was constructed using the neighbor-joining method, and the relative stability of the branches was evaluated by bootstrap analysis of 1,000 datasets using MEGA11 software (Tamura et al., 2021).

#### 2.1.2 Phosphate solubilization

To quantify phosphate solubilization, bacterial strains were inoculated into a National Botanical Research Institute Phosphorus (NBRIP) medium consisting of 10 g glucose, 5 g MgCl_2_∙6H_2_O, 0.25 g MgSO_4_∙7H_2_O, 0.2 g KCl, 0.1 g (NH_4_)_2_SO_4_, and 5 g tricalcium phosphate as the sole P source in 1 L of distilled water (Olsen et al., 1954). Cultured bacterial strains (0.3 mL) were inoculated into 30 mL of NBRIP medium in Erlenmeyer flasks. Controls were prepared using the NBRIP medium only. After incubation for 7 days at 30°C and 150 rpm, the culture medium was centrifuged to quantify soluble phosphorus using the MARS 6 microwave instrument from CEM (Kamp-Lintfort, Germany). For the digestion, 5 mL of the supernatant was transferred to a 110 mL vessel, and 15 mL of 70% nitric acid solution was added. The mixture was subjected to a temperature–time ramp, reaching a final temperature of 180°C within 15 min, and this temperature was maintained for 20 min to ensure complete acid digestion. Following digestion, the 70% nitric acid was evaporated with heat, and the residue was diluted with 3% nitric acid solution for analysis. The diluted sample was analyzed using inductively coupled plasma mass spectrometry (ICP-MS) on an iCAP-RQ instrument (Thermo Fisher Scientific, Waltham, MA, United States) equipped with an ASX-560 autosampler (TELEDYNE, Englewood, CO, United States). The analysis utilized the ^31^P isotope for phosphorus quantification. A kinetic energy discrimination mode with helium gas was employed to mitigate interference from ^14^N^16^OH^+^ polyatomic ions (Fleischer et al., 2014).

#### 2.1.3 Nitrogen fixation

Molecular nitrogen fixation was assessed using a nitrogen-free medium (Nfb) and the phenate method to detect NH_4_-N production (Ahmad et al., 2013). A bacterial colony was inoculated into 25 mL of Nfb and cultured at 30°C for 7 days. The culture medium was centrifuged at 4°C and 8,000 rpm for 20 min to obtain a supernatant. The supernatant was then diluted 10 times into 25 mL portions and added with 1 mL of phenol, 1 mL of sodium nitroprusside, and 2.5 mL of oxidizing solution. The mixed solution was incubated for 1 h, and the absorbance was measured at 640 nm to quantify NH_4_-N production using an ammonium standard solution (0.1, 0.2, 0.5, and 1 mg N/L) for calibration. Distilled water was used as a blank.

#### 2.1.4 IAA production

The colorimetric determination of IAA was performed as previously described (Meudt and Gaines, 1967). The bacterial strains were cultured in DF medium [2 g (NH_4_)_2_SO_4_, 1 g KH_2_PO_4_, 15 g Na_2_HPO_4_·7H_2_O, 0.2 g MgSO_4_·7H_2_O, 1 mg FeSO_4_·7H_2_O, 10 μg H_3_BO_3_,11 μg MnSO_4_·H_2_O, 125 μg ZnSO_4_·7H_2_O, 78 μg CuSO_4_·5H_2_O, and 17 μg Na_2_MoO_4_·2H_2_O per liter] supplemented with 0.5 g tryptophan and then incubated at 30°C and 180 rpm for 5 days. After centrifugation at 5000 rpm for 20 min, 1 mL of the supernatant was mixed with 2 mL of modified Salkowski reagent (50 mL of perchloric acid and 1 mL of 0.05 M FeCl_3_) and left in the dark at 24°C for 30 min. The optical density (OD) was measured at 530 nm using microplate reader (SPARK, TECAN, Männedorf, Switzerland), and the IAA concentration was calculated from a standard curve prepared using IAA solutions ranging from 0 to 100 ng/mL.

#### 2.1.5 Siderophore production

Siderophore production was assessed using King’s B broth (20 g protease peptone, 10 mL glycerol, 1.5 g KH_2_PO_4_, and 1.5 g MgSO_4_·7H_2_O in 1 L). The medium was iron-deficient to induce siderophore synthesis. For quantitative analysis, 0.4 mL of the bacterial cultures were inoculated into 40 mL of King’s B broth and incubated at 30°C for 4 days as previously described (Nagarajkumar et al., 2004; Cherif-Silini et al., 2016). After centrifugation at 5000 rpm for 30 min, the supernatant was adjusted to pH 2.9, mixed with an equal volume of ethyl acetate, shaken for 30 min, and then recentrifuged at 5,000 rpm for 30 min. The upper ethyl acetate layer was collected and mixed with Hathway reaction solution (1 mL of 0.1 M FeCl_3_ in 0.1 N HCl added to 100 mL distilled water plus 1 mL of potassium ferricyanide). After 20 min, the absorbance was measured at 700 nm using microplate reader (SPARK, TECAN). Siderophore production was quantified based on the measurements, and a standard curve was prepared using dihydroxybenzoic acid.

#### 2.1.6 ACC deaminase production

The ACC deaminase activity was evaluated using a previously described method (Misra and Chauhan, 2020), which measures α-ketobutyrate production resulting from ACC cleavage by ACC deaminase. Briefly, to measure ACC deaminase production, 20 μL of 0.5 M ACC was added to 0.2 mL of cell pellets obtained by inoculating the isolated strain into DF salts minimal medium containing 3 mM ACC as the sole nitrogen source [replacing (NH_4_)_2_SO_4_]. The reaction was allowed to proceed for 15 min. After the addition of 0.56 M HCl and 2,4-dinitrophenylhydrazine reagent (0.2% 2,4-dinitrophenylhydrazine in 2 M HCl), the mixture was stirred for 30 min and then added with 2 mL of 2 N NaOH. Absorbance was measured at 540 nm, and ACC deaminase activity was quantified by comparing the absorbance with a standard curve of known α-ketobutyrate concentrations.

#### 2.1.7 Bacterial growth and exopolysaccharide formation

Bacterial growth was evaluated at different water potentials of 0, −0.15, and −0.49 MPa, which were generated using LB liquid medium supplemented with polyethylene glycol (PEG) 6,000 at concentrations of 0, 10, and 20%, respectively. The selected strains were inoculated into the medium at an initial OD of 0.01 and then cultured at 30°C with shaking at 180 rpm for 48 h. Bacterial growth was monitored by measuring the OD_600nm_ using microplate reader (SPARK, TECAN). Growth at 0 MPa was considered 100%, whereas growth at −0.15 and −0.49 MPa was expressed as a percentage relative to that at 0 MPa.

The production of EPS (total biopolymers) at different water potentials (0, −0.15, and −0.49 MPa) was determined in broth cultures of the same strains. After inoculation into nutrient broth supplemented with 5% glucose and incubation at 30°C and 120 rpm for 40 h, the supernatant was collected by centrifugation at 12,000 rpm for 20 min at 4°C. Acetone was added to the supernatant in a ratio of 1:2 (v:v), and the mixture was allowed at 4°C for 24 h to precipitate the EPS. The precipitate was filtered through filter paper and dried at 24°C, and the weight of the filter paper was measured. The blank was measured using untreated filter paper for value calculation. Each treatment was performed in triplicate in two biological experiments, including control samples inoculated with *B. megaterium* ATCC14581.

#### 2.1.8 Whole genome sequencing, gene prediction, and annotation

The genomes of CACC109 and CACC119 were assembled *de novo* from PacBio sequencing data by CJ Bioscience, Inc. (South Korea). Contigs were assembled and circularized using SMRT Link (Pacific Biosciences, Menlo Park, CA, United States) and Circlator 1.4.0 (Sanger Institute, Essex, United Kingdom). Gene prediction and functional annotation were performed using the EzBioCloud genome database, Prodigal 2.6.2 (CDSs) (Hyatt et al., 2010), tRNAscan-SE 1.3.1 (tRNA) (Schattner et al., 2005), and Rfam 12.0, (rRNA and other non-coding RNAs) (Nawrocki et al., 2015). CRISPR detection was conducted using PilerCR 1.06 (Edgar, 2007) and CRT 1.2 (Bland et al., 2007). Functional classification was performed using EggNOG 4.5.^2^ Additional annotation was achieved by comparing the predicted CDSs with the Swissprot (The UniProt Consortium, 2015), KEGG (Kanehisa et al., 2014), and SEED databases (Overbeek, 2005) using the UBLAST program (Edgar, 2010).

### 2.2 Effect of *B. megaterium* CACC109 and CACC119 on drought tolerance

#### 2.2.1 Effects of bacterial strains on rice germination and growth under osmotic-stressed and non-stressed conditions

Germination and seedling growth assays were performed on rice seeds to evaluate the efficacy of the isolated bacterial strains in improving osmotic stress tolerance. Saechungmu rice (*Oryza sativa* L.) seeds were surface sterilized with 2% sodium hypochlorite and 70% ethanol and then washed multiple times with distilled water. The seeds were filled to the 20 mL mark in a 50 mL conical tube and either 1 × 10^7^ CFU (colony-forming unit)/mL of bacterial suspension or 0.85% saline (mock) was added up to the 40 mL mark, ensuring the seeds were completely submerged. The seeds were then gently shaken for 8 h. Twenty seeds from each treatment were evenly distributed among three Petri dishes each containing Murashige & Skoog (MS) medium (DUCHEFA BIOCHEMIE, The Netherlands) with different water potentials (0, −0.15, and −0.49 MPa) achieved with PEG 6000. Germination was carried out at 27°C in the dark for 5 days, and then the germination rate, root length, and growth parameters were measured. The germination rate was calculated by dividing the number of germinated seeds by the total number of seeds and expressed as a ratio (Islam et al., 2016).

A similar assay was conducted using an iso-osmotic solution of mannitol to assess the effect of the isolated bacterial strains on osmotic stress tolerance. Seeds treated with bacteria or mock solution were germinated in a dark box at 27°C for 3 days. Uniformly germinated seeds were then transferred to MS agar supplemented with 0, 150, and 200 mM mannitol and grown under a 12-h light–dark cycle at 27°C for 3 days. Seedling root length was measured after 3 days. Each assay consisted of 10 seeds arranged in Petri dishes, with three replicates per treatment, and securely sealed with Parafilm.

#### 2.2.2 Experimental design for plant growth and drought study

To study the effects of bacterial strains on rice growth and various physiological, molecular, and biochemical responses under drought and non-drought conditions, Saechungmu rice seeds were germinated at 27°C in the dark for 3 days. Uniformly germinated seedlings, 5 or 30, were selected and planted in regular or Wagner pots, respectively, filled with autoclaved potting soil for rice plants (Punong, Gyeongbuk, South Korea). At 7 days post-planting, the plants were treated weekly for 4 weeks with either 5 mL of 1 × 10^7^ CFU/mL *B. megaterium* CACC109, 1 × 10^7^ CFU/mL *B. megaterium* CACC119, or 0.85% saline (mock) per plant. A 1,000-fold dilution of Hyponex fertilizer was applied to the plants at 10 days post-planting. The plants were maintained in a growth chamber with a constant temperature of 27°C, a 12-h light–dark cycle, and approximately 70% relative humidity. After 6 weeks of growth, watering was halted for half of the mock-, CACC109-, and CACC119-treated rice plants, while the remaining plants continued to grow under well-watered conditions, serving as the control group. Soil moisture content was monitored daily using a moisture analyzer MA37 (Sartorius, Göttingen, Germany) to ensure a consistent water retention rate of 20–40% throughout the drought.

Chlorophyll content was assessed by measuring 20 leaves from mock-, CACC109-, and CACC119-treated independent plants in both the well-watered and water-stressed groups using a SPAD-502 meter (Konica Minolta, Tokyo, Japan). The height, weight, and water retention of 20 plants from each treatment group were assessed 15 or 37 days after inducing drought in the well-watered and water-stressed groups. Each plant, including its roots, was weighed to determine the fresh weight, followed by drying at 65°C for 3 days and subsequent weighing to obtain the dry weight. Water retention for each plant was calculated as the difference between the fresh and dry weights.

For gene expression analysis, leaf samples were collected from the mock-, CACC109-, and CACC119-treated plants grown under well-watered and water-stressed conditions at three time intervals (early, 10 days; middle, 20 days; and late, 35 days post-drought). For each treatment group, four biological replicates were sampled, with each biological replicate being a pool of three leaf tissues from independent plants. The collected samples were immediately flash-frozen in liquid nitrogen and stored at −80°C.

#### 2.2.3 Estimation of proline content

Total proline accumulation in the leaf samples was quantified as previously described (Bates et al., 1973). Briefly, 150 mg of leaf samples were ground with liquid nitrogen and homogenized in 2 mL of 3% sulfosalicylic acid. After centrifugation at 12,000 rpm for 10 min, the supernatant was transferred to a new tube. Then, 0.5 mL of the supernatant was mixed with 0.5 mL of ninhydrin and 0.5 mL of glacial acetic acid in a 15 mL conical tube. The mixture was boiled for 45 min and immediately cooled on ice for 30 min. The samples were mixed with 1.5 mL of toluene and centrifuged at 700 rpm for 5 min to separate the total proline. Total proline content was measured at 520 nm against a blank using a standard curve prepared with proline.

#### 2.2.4 Total RNA extraction and gene expression analysis

Total RNA from leaf tissue was extracted using TRIzol reagent (Thermo Fisher Scientific, Waltham, MA, United States). The RNA was treated with DNase I (Promega, Madison, WI, United States), cleaned using UltraPure™ Phenol:Chloroform:Isoamyl Alcohol (25, 24:1, v/v) (Thermo Fisher Scientific), and checked for quality and quantity using NANODROP ONE (Thermo Fisher Scientific). For each line, cDNA was synthesized from 2 μg of total RNA using M-MLV reverse transcriptase (Promega). Quantitative PCR analyses were conducted on a CFX96 real-time PCR system (Bio-Rad Laboratories, Hercules, CA, United States) using SYBR Green Supermix (containing hot-start iTaq DNA polymerase, dNTPs, MgCl2, SYBR^®^ Green I dye, enhancers, and stabilizers from Bio-Rad Laboratories) and gene-specific primers. Samples were prepared in triplicate, with a total reaction volume of 20 μL containing the following components: 2 μL of cDNA, 1 μL of each primer (forward and reverse, at 10 pM each), 10 μL of SYBR Green Supermix, and 6 μL of double-distilled water. Amplification reactions were initiated with a denaturation step at 95°C for 30 s, followed by 40 cycles of 95°C for 5 s, 60°C for 10 s, and 72°C for 20 s. Melting curves were recorded over a temperature range of 65°C to 95°C in 0.5°C increments. Rice *Actin* served as an endogenous control for normalization. Expression levels were determined using the comparative cycle threshold (Ct) method. Details of the quantitative reverse transcription polymerase chain reaction (qRT-PCR) primers used are listed in Supplementary Table S1.

#### 2.2.5 Analysis of chemical properties of soil

Soil samples (200 g each) were collected from each pot after the drought stress experiment. The samples were air-dried at room temperature, crushed to pass through a 2 mm mesh sieve, and thoroughly mixed. The pH was measured in distilled water at a ratio of 1:5 (soil sample: distilled water) using a glass electrode pH meter. Exchangeable cations from the soil were extracted with 0.05 N NH_4_OAc (pH 7.0) and determined using iCAP™ RQplus ICP-MS (Thermo Fisher Scientific). Available phosphoric acid was extracted following the Lancaster method and determined using iCAP™ RQplus ICP-MS (Thermo Fisher Scientific).

### 2.3 Statistical analysis

Statistical analyses were performed using the JMP Pro 16 software package. Two-way analyses of variance (ANOVA) with Tukey’s honest significant difference (HSD) test were used to assess the statistical significance of the bacterial growth, EPS production, seed germination, plant height, root length, chlorophyll content, proline production, and gene expression assay. Students’ *t*-test was used to compare significant differences in plant growth-promoting traits (phosphate solubility, nitrogen fixation, IAA productivity, siderophore productivity, and ACC deaminase activity), plant weight, water retention, survival rate under salinity stress, and soluble organic components in soil. The significance level was determined at a *p* < 0.05.

## 3 Results

### 3.1 PGPR isolated and screened from the ginseng rhizosphere

**Figure 1:**
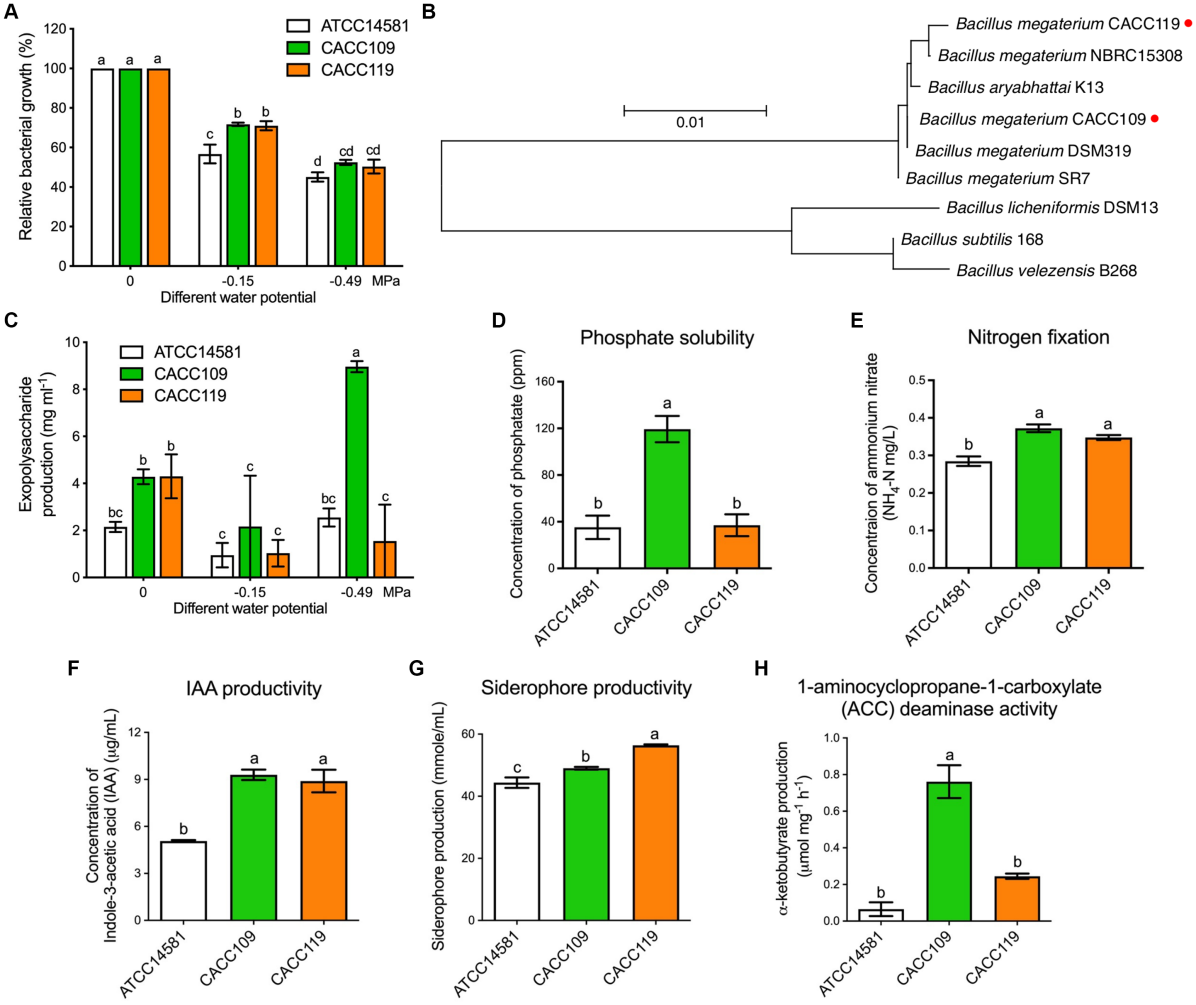
Isolation, characterization, and plant growth-promoting properties of water stress-tolerant *Bacillus megaterium* strains CACC109 and CACC119. **(A)** Relative growth rates of *B. megaterium* strains CACC109 and CACC119 *in vitro* at different water potentials. Growth was measured 24 h after incubation. Growth at 0 MPa was converted to 100%, and the growth values at −0.15 and −0.49 MPa were expressed as a percentage relative to that at 0 MPa. Data are expressed as the mean ± SD of three biological replicates. Different letters indicate significant differences among the bacterial strains in the various water potentials (*p* < 0.05, two-way ANOVA and Tukey’s test). *B. megaterium* ATCC14581 was used as a control. **(B)** Evolutionary relationships of CACC109 and CACC119 with other known *Bacillus* strains. Sequences were aligned through “Clustal W” using MEGA 11. The maximum-likelihood method with the Tamura-Nei model was used to construct the phylogenetic tree based on 16S rRNA gene nucleotide sequences. Bootstrapped consensus tree derived from 1,000 replicates. Evolutionary analysis was performed in MEGA 11. Red dots indicate the positions of CACC109 and CACC119. **(C)** EPS production of CACC109 and CACC119 *in vitro* at different water potentials 40 h after inoculation. Bars represent the mean ± SE of three biological replicates. Different letters indicate significant differences among the bacterial strains in the various water potentials (*p* < 0.05, two-way ANOVA and Tukey’s test). **(D)** Phosphate solubilization, **(E)** nitrogen fixation, **(F)** indole-3-acetic acid (IAA) production, **(G)** siderophore production, and **(H)** 1-aminocyclopropane-a-carboxylate (ACC) deaminase activity. Data in **(D–H)** are expressed as mean ± SD of three biological replicates. Different letters indicate significant differences (*p* < 0.05, Student’s *t*-test). *B. megaterium* ATCC14581 type strain was used as a control. *B. megaterium* ATCC14581 was used as a control. Accession numbers: *Bacillus megaterium* CACC119 (OR915158), *Bacillus megaterium* NBRC15308 (CP009920), *Bacillus aryabhattai* K13 (CP024035), *Bacillus megaterium* CACC109 (CP117689), *Bacillus megaterium* DSM319 (CP001982), *Bacillus megaterium* SR7 (CP022674), *Bacillus licheniformis* DSM13 (AE017333), *Bacillus subtilis* 168 (CP053102), *Bacillus velezensis* B268 (CP053764).

Five ginseng rhizosphere soil samples were collected from ginseng cultivation fields in South Korea to isolate PGPR with osmotic stress tolerance. A total of 31 isolates were obtained from the samples and identified based on their 16S rRNA gene sequences (Supplementary Table S2). Subsequently, the isolates were screened for osmotic stress tolerance in LB medium supplemented with PEG 6000. Two isolates exhibited better growth than the *B. megaterium* type strain ATCC14581 under osmotic stress (Figure 1A), indicating their efficacy as stress-tolerant isolates. Therefore, these strains were selected for further studies.

Identification of these two isolates by 16S rRNA gene sequencing revealed that they belonged to *B. megaterium* and thus were designated as *B. megaterium* CACC109 and CACC119. The obtained sequences of CACC109 and CACC119 were submitted to NCBI GenBank under accession numbers CP117689 and OR915158, respectively. A phylogenetic analysis was conducted using 16S rRNA gene sequences. The sequences of CACC109 and CACC119, along with their closest relatives from NCBI GenBank, were used to construct a phylogenetic tree with the maximum-likelihood method. The analysis showed that CACC109 was most closely related to *B. megaterium* DSM319, whereas CACC119 was most closely related to *B. megaterium* NBRC15308 (Figure 1B).

The EPS production of the bacterial strains subjected to different levels of osmotic stress induced by PEG6000 was quantified. Under non-osmotic stress, CACC109 and CACC119 produced 4.28 and 4.3 mg mL^−1^ EPS, respectively, which were significantly higher than the 2.15 mg mL^−1^ EPS produced by ATCC14581 (Figure 1C). However, EPS production decreased under −0.15 MPa osmotic stress. Remarkably, CACC109 demonstrated the highest EPS production, reaching 8.96 mg mL^−1^ at −0.49 MPa, whereas CACC119 exhibited substantially lower levels than CACC109 and similar levels to ATCC14581 (Figure 1C).

Analysis of the PGP properties of osmotic-tolerant strains CACC109 and CACC119 revealed that CACC109 had a significant ability to solubilize phosphate, with a maximum soluble P_2_O_5_ concentration of 119 μg/mL, whereas CACC119 did not (Figure 1D). In terms of nitrogen fixation, CACC109 and CACC119 produced 0.372 and 0.347 μg/mL NH_4_, respectively (Figure 1E). In terms of IAA production, CACC109 and CACC119 produced significantly higher concentrations of indole compounds than ATCC14581 (Figure 1F). Although both strains tested positive for siderophore production, CACC119 exhibited significantly greater production of siderophores than CACC109 (Figure 1G). Meanwhile, CACC109 exhibited significant ACC deaminase activity, whereas CACC119 did not (Figure 1H).

### 3.2 Effects of CACC109 and CACC119 on seed germination and root growth

**Figure 2:**
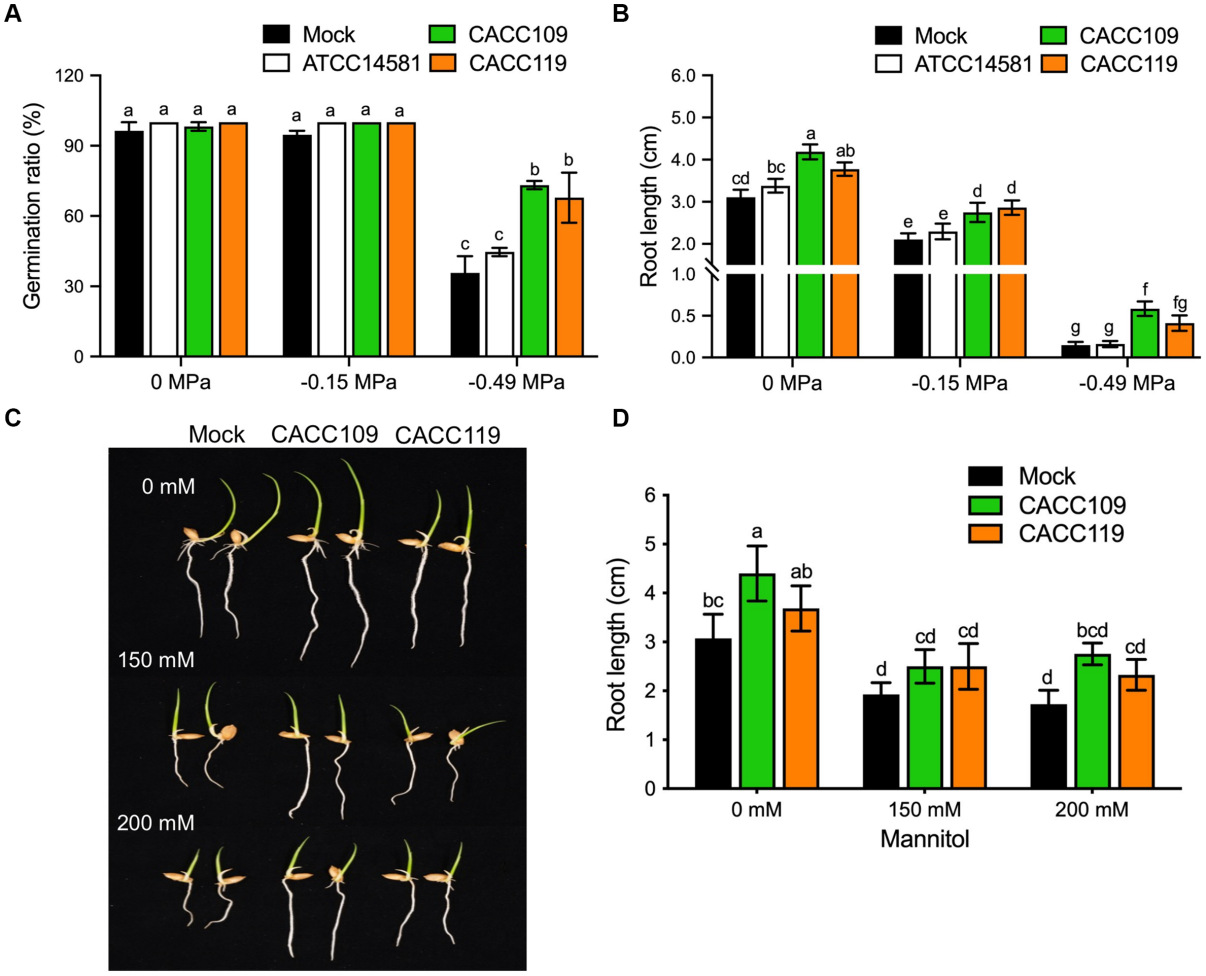
Effects of CACC109 and CACC119 on the osmotic stress resistance in rice seedlings. **(A)** Germination rate and **(B)** root length in rice seeds treated with mock, CACC109, or CACC119 at different water potentials. **(C)** Root growth of rice seedlings treated with mock, CACC109, or CACC119 grown in Murashige & Skoog (MS) media containing 0, 150, and 200 mM mannitol for 3 days. **(D)** Root length in **(C)** (*n* > 30). In **(A,B)**, seeds were soaked in mock, CACC109, or CACC119 for 8 h and incubated at 30°C for 5 days under different water potentials created with PEG6000. In **(C,D)**, for the mannitol assays, rice seeds were soaked in mock, CACC109, or CACC119 for 10 h, germinated in the magenta box, and similar-sized 3-day-old seedlings were transferred to MS media containing 0, 150, and 200 mM mannitol. Images and measurements were taken 3 days after growth on the MS medium. Assays were performed using 1 × 10^7^ CFU/mL of CACC109 and CACC119. Data represent the mean ± SE of three independent experiments. Different letters indicate significant differences among the treatments in the various osmotic stresses (*p* < 0.05, two-way ANOVA and Tukey’s test).

The effects of CACC109 and CACC119 on the germination and root growth of rice seeds were examined under osmotic-stressed and non-stressed conditions. Under non-stressed conditions (0 MPa), the CACC109-, CACC119-, ATCC14581-, and mock-treated seeds showed identical germination ratios, with the maximum germination observed on day three (Figure 2A). At −0.15 MPa, no significant differences in germination rates were observed among the treatment groups (Figure 2A). However, the germination rates of the CACC109- and CACC119-treated seeds were significantly higher than those of the mock- and ATCC14581-treated lines at −0.49 MPa, indicating that both PGPR strains supported seed germination in rice under osmotic stress challenge (Figure 2A). In addition, the CACC109- and CACC119-treated seeds had significantly greater root growth than the mock- and ATCC14581-treated seeds under osmotic-stressed and non-stressed conditions (Figure 2B).

To validate the effects of CACC109 and CACC119 on rice root growth, we germinated rice seeds and selected evenly germinated seedlings. The seedlings were then exposed to different concentrations of mannitol to induce osmotic stress (Claeys et al., 2014). The root lengths of the mock-, CACC109-, and CACC119-treated seedlings gradually decreased with increasing osmotic stress. This effect is similar to the observed effects of PEG-induced osmotic stress. In the absence of osmotic stress, the CACC109- and CACC119-treated seedlings had significantly longer roots than the mock-treated seedlings (Figures 2C,D). When exposed to 150 or 200 mM mannitol, the CACC109- and CACC119-treated seedlings had significantly longer roots than the mock-treated seedlings, indicating that both CACC strains promoted root growth of the rice seedlings under normal and drought conditions (Figures 2C,D). Collectively, these data indicated that CACC109 and CACC119 enhanced osmotic stress tolerance, promoted seed germination, and facilitated root development in rice seedlings.

### 3.3 Effects of CACC109 and CACC119 on plant growth and drought resistance

**Figure 3:**
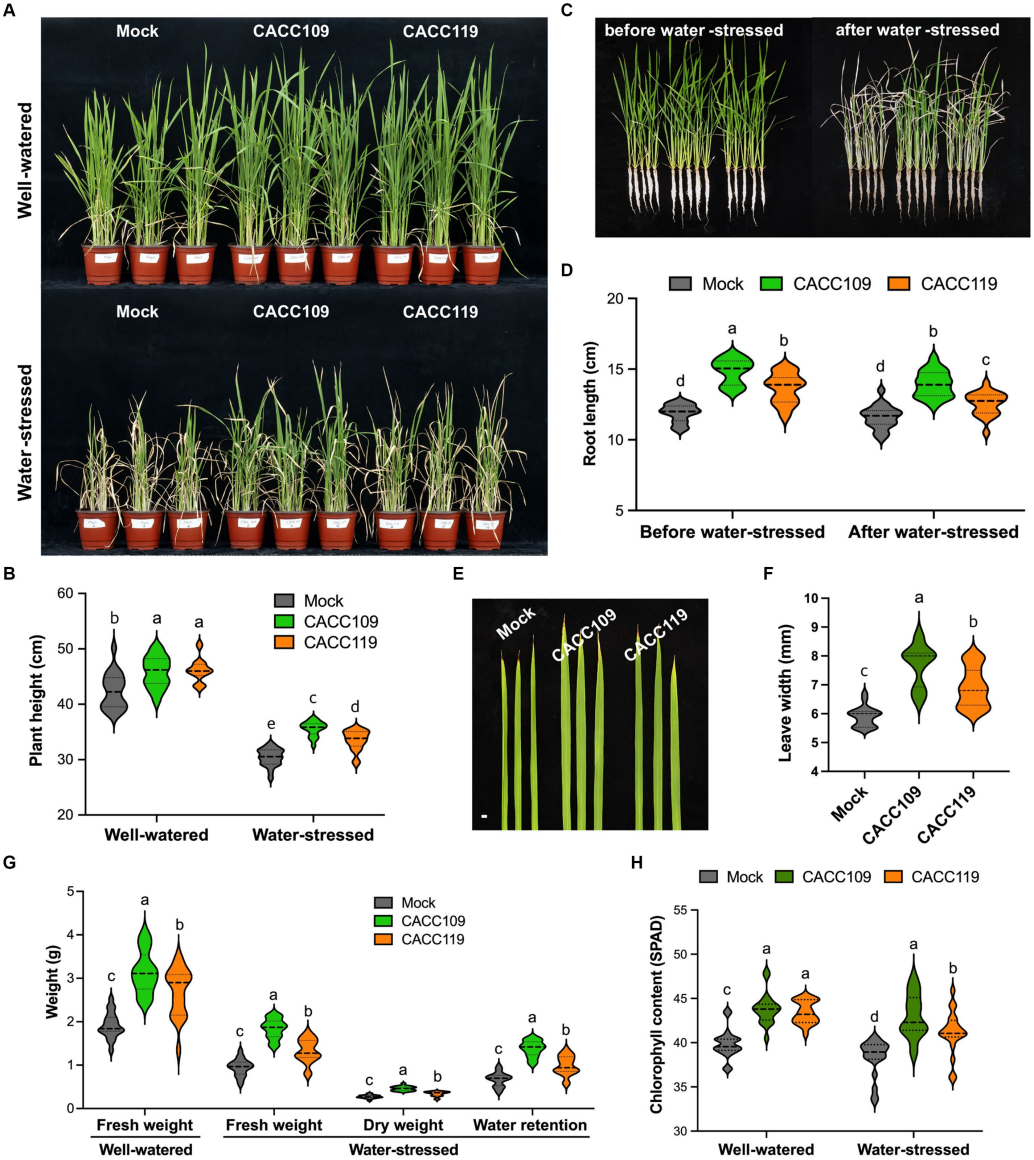
Growth promotion and drought resilience in rice by CACC109 and CACC119. **(A)** Rice plants treated with mock, CACC109, or CACC119 under well-watered and water-stressed conditions. The 6-week-old mock-, CACC109-, or CACC119-treated plants (25 plants for each line) were subjected to drought with 20–40% soil water potential for 15 days. **(B)** Height of rice plants treated with mock, CACC109, or CACC119 under well-watered and water-stressed conditions. **(C)** Aboveground shoot and root lengths of rice plants treated with mock, CACC109, or CACC119 before water stress (6 weeks old) or after water stress (15 days under drought). **(D)** Root length in **(C)**. **(E)** Flag leaf morphology of 7-week-old rice plants treated with mock, CACC109, or CACC119 under well-watered conditions, bar = 5 mm. **(F)** Leaf width in **(E). (G)** Fresh weight of mock-, CACC109-, or CACC119-treated rice plants grown under well-watered conditions for 8 weeks. Fresh weight, dry weight, and water retention (fresh weight – dry weight) of mock-, CACC109-, or CACC119-treated rice plants after 15 days of drought. **(H)** Chlorophyll content of mock-, CACC109-, or CACC119-treated rice plants grown under well-watered or water-stressed conditions. In **(B–H)**, values are means ± SD (*n* = 20, individual plants). Different letters indicate significant differences (*p* < 0.05, two-way ANOVA and Tukey’s test in **B,D,H**; Student’s *t*-test in **F,G**). Control plants (mock) were treated with the same amount of saline solution as a negative control. Chlorophyll and height were measured on mature leaves. The results presented here are representative of three independent experiments.

We investigated the effects of PGPR CACC109 and CACC119 on rice growth and drought stress resilience. Under well-watered conditions, the CACC109- and CACC119-treated rice plants had significantly greater shoot and root growth than the mock-treated plants (Figures 3A–D). Treatment with CACC109 and CACC119 notably increased the rice leaf width (Figures 3E,F). The CACC109- and CACC119-treated plants exhibited 64 and 40% higher shoot weight, respectively, than the mock-treated plants (Figure 3G). Compared with the mock treatment, CACC109 considerably increased root biomass due to axial and lateral root development, whereas CACC119 had a less pronounced effect (Supplementary Figure S1). These results indicated that CACC application significantly promoted plant growth in rice.

After 15 days of water stress, all mock-treated rice plants displayed typical severe dehydration symptoms with significant wilting, and only a few leaves remained green. By contrast, the leaves of most CACC109-treated plants appeared less dehydrated and remained green. The CACC119-treated plants exhibited an intermediate drought stress phenotype relative to the mock- and CACC109-treated plants (Figure 3A). Additionally, water stress caused growth retardation in the mock- and PGPR-treated plants, although this effect was more pronounced in the mock-treated plants (Figures 3A–D). Under drought, the CACC109- and CACC119-treated plants had greater shoot weights than the mock-treated plants (Figure 3G). Under water-stressed conditions, the CACC109-treated plants showed significantly higher shoot dry weight than the mock-inoculated plants. However, no significant difference in shoot dry weight was found between the CACC119- and mock-treated plants (Figure 3G). Drought is typically characterized by decreased water and chlorophyll contents (Bhandari et al., 2023). The CACC109- and CACC119-treated plants had higher water retention than the mock-treated plants (Figure 3G). After 10 days of drought, the chlorophyll content in the mock-treated plants decreased by 7%, whereas those in the CACC109- and CACC119-treated plants decreased by only 3 and 3.5%, respectively, compared with their respective controls (Figure 3H). These data were consistent with the phenotypes of rice plants under drought in each treatment group. Taken together, these results indicated that CACC109 and CACC119 significantly improved the growth and the drought tolerance of rice plants.

### 3.4 Evaluation of CACC109 and CACC119 on drought tolerance in rice

Additional pot experiments were conducted to evaluate the effects of PGPR CACC109 and CACC119 on the drought tolerance in rice plants. These studies were conducted using a large number of rice plants grown in Wagner pots to mimic conditions in rice fields. We supplied CACC109 and CACC119 in the same manner as in the small-pot test and carefully maintained water levels throughout the experiment to simulate rice field conditions. To investigate the interaction between plants and PGPR, we collected leaf tissues from the CACC109-, CACC119-, and mock-treated plants under non-stressed and water-stressed conditions at different stages of drought (early, 10 days; middle, 20 days; and late, 35 days) to examine physiological traits and gene expression.

**Figure 4:**
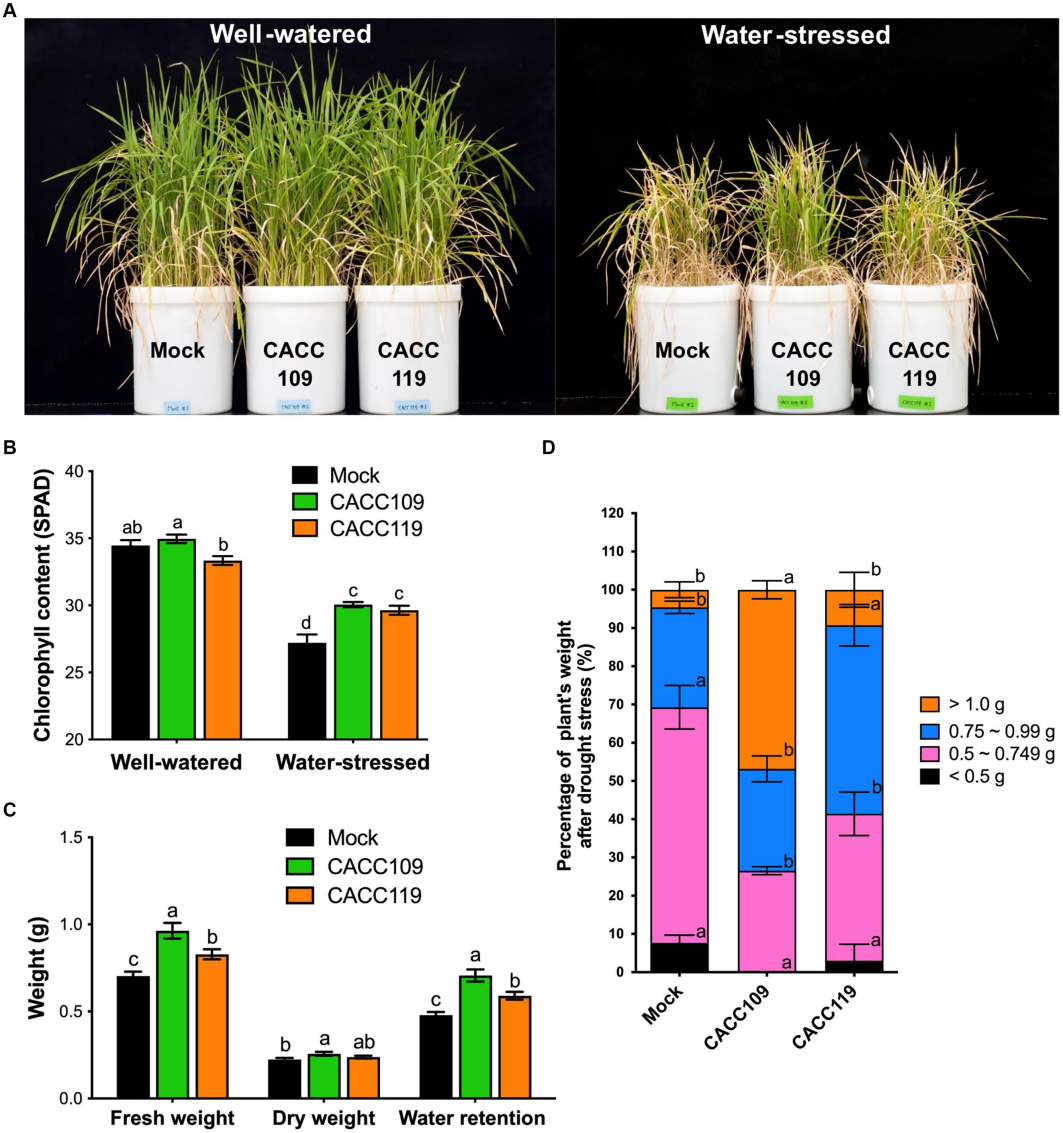
Enhanced drought tolerance in rice plants treated with CACC109 and CACC119. **(A)** Representative images of rice plants treated with mock, CACC109, or CACC119 under both well-watered and water-stressed conditions. **(B)** The chlorophyll content of rice plants grown under well-watered or water-stressed conditions. **(C)** Fresh weight, dry weight, and water retention (fresh weight-dry weight) of rice plants under drought. **(D)** Individual fresh weights of rice under water-stressed conditions. In **(B–D)**, values are means ± SD (*n* = 30, individual plants). Different letters represent significant differences (*p* < 0.05, two-way ANOVA and Tukey’s test in **B**; Student’s *t*-test in **C,D**). Control plants (mock) were treated with equal volumes of saline solution as negative control. The soil water content in the water-stressed group throughout the drought stress period was determined using a moisture analyzer MA37 and calculated as a percentage of field capacity. The experiment was performed two times.

After 37 days of water deprivation, all mock-treated plants exhibited severe drought symptoms, characterized by significant wilting and a few leaves remaining green (Figure 4A), whereas the CACC109-treated plants appeared less dehydrated and remained green. The CACC119-treated plants exhibited an intermediate drought stress phenotype compared with the mock- and CACC109-treated plants (Figure 4A). Similar results were observed in these experiments, showing that the application of CACC109 and CACC119 increased the total chlorophyll content, fresh weight, and water retention in the rice plants under drought (Figures 4B,C). The weights of the drought-stressed plants were categorized (Figure 4D). In the mock treatment group, 69.27% of the plants weighed less than 0.75 g, whereas 26.13% weighed within 0.75–0.99 g, with only 4.6% weighing over 1.0 g. By contrast, 26.53% of the CACC109-treated plants weighed within 0.5–0.749 g, 26.64% within 0.75–0.99 g, and 46.83% exceeded 1 g. Meanwhile, 41.42% of the CACC119-treated plants weighed less than 0.749 g, 49.29% weighed within 0.75–0.99 g, and 9.29% exceeded 1 g (Figure 4D). Overall, these data confirmed that CACC109 and CACC119 enhanced the drought tolerance in rice plants.

### 3.5 Enhanced proline accumulation and gene expression by CACC109 and CACC119 under drought stress

**Figure 5:**
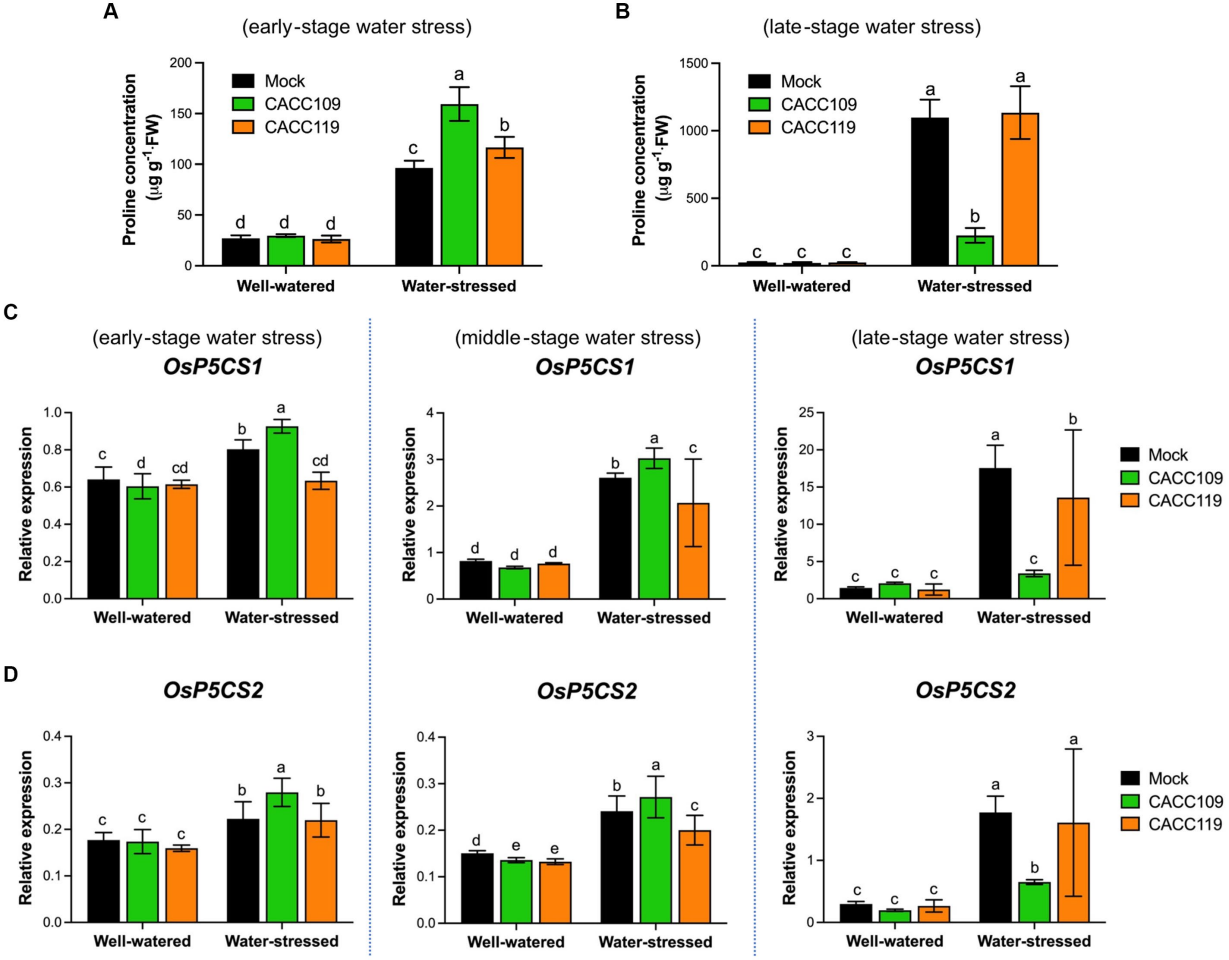
Effects of CACC109 and CACC119 on drought-induced proline production. **(A,B)** Proline content measured in the leaves of mock-, CACC109-, and CACC119-treated plants with or without drought stress in accordance with the method described by (Lugtenberg and Kamilova, 2009). Proline levels were quantified with four biological replicates at **(A)** 10 days and **(B)** 35 days post-drought. **(C,D)** Expression levels of *pyrroline-5-carboxylate synthetas*e 1 (*P5CS1*) and *P5CS2* in rice leaves grown with mock, CACC109, or CACC119 under well-watered and water-stressed conditions. P5CS catalyzes the first step of proline biosynthesis from glutamate. Six-week-old plants were subjected to drought by maintaining soil water potential at 20–40% for 37 days. Leaf tissues were collected from three independent plants treated with mock, CACC109, or CACC119 in each of the two pots under well-watered and water-stressed conditions at 10 (early), 20 (middle), and 35 (late) days after drought treatment. The expression levels of *OsP5CS1* and *OsP5CS2* were estimated by quantitative real-time PCR, and *OsActin* was used as an internal control for normalization. Values are mean ± SD of two independent experiments (*p* < 0.05, two-way ANOVA and Tukey’s test).

Plants under drought and high salinity often accumulate proline to enhance stress adaptation (Hayat et al., 2012). We evaluated the proline levels in the leaf tissues from the CACC109-, CACC119-, and mock-treated plants under well-watered and water-stressed conditions. No significant difference in proline content was found between the PGPR- and mock-treated plants under normal growth conditions (Figures 5A,B). All plants subjected to water stress displayed significantly higher proline concentrations than the well-watered control group (Figures 5A,B), indicating that the drought treatment was successful. Interestingly, at the early stage of water stress, the CACC109- and CACC119-treated plants had significantly higher leaf tissue proline concentrations than the mock-treated plants (Figure 5A). As the water stress continued, all plants accumulated more proline, but the mock- and CACC119-treated plants showed significantly higher proline accumulation than the CACC109-treated plants (Figure 5B). This result indicated that the mock- and CACC119-treated plants experienced relatively stronger water stress intensity than the CACC109-treated plants.

We also investigated the time-course expression patterns of the proline biosynthesis genes *Delta 1-Pyrroline-5-carboxylate synthetase 1* and *2* (*P5CS1* and *P5CS2*) in the CACC109-, CACC119-, and mock-treated plants grown under well-watered and water-stressed conditions. As expected, no significant differences in the transcript levels of *P5CS1* and *P5CS2* were found among the treatment groups under well-watered conditions (Figures 5C,D). The expression of *P5CS1* and *P5CS2* was upregulated by water stress in all treated rice plants. Specifically, the expression levels of *P5CS1* and *P5CS2* were higher in the CACC109-treated plants than in the mock- and CACC119-treated plants at the early and middle stages of water stress but lower at the late stage of water stress (Figures 5C,D). The observed trends in the transcript levels of *P5CS1* and *P5CS2* were consistent with proline accumulation. These data suggested that rice produced proline in response to water stress and that inoculation with the PGPR further improved stress tolerance in rice plants by enhancing proline accumulation.

### 3.6 Effects of CACC109 and CACC119 on the expression of genes responsible for eliminating reactive oxygen species

Under drought, reactive oxygen species (ROS) trigger defense mechanisms associated with ABA and Ca^2+^ flux (Mahmood et al., 2019). However, excessive ROS accumulation leads to cell death through oxidative damage, emphasizing the necessity of ROS-detoxifying systems for plant resilience to drought.

**Figure 6:**
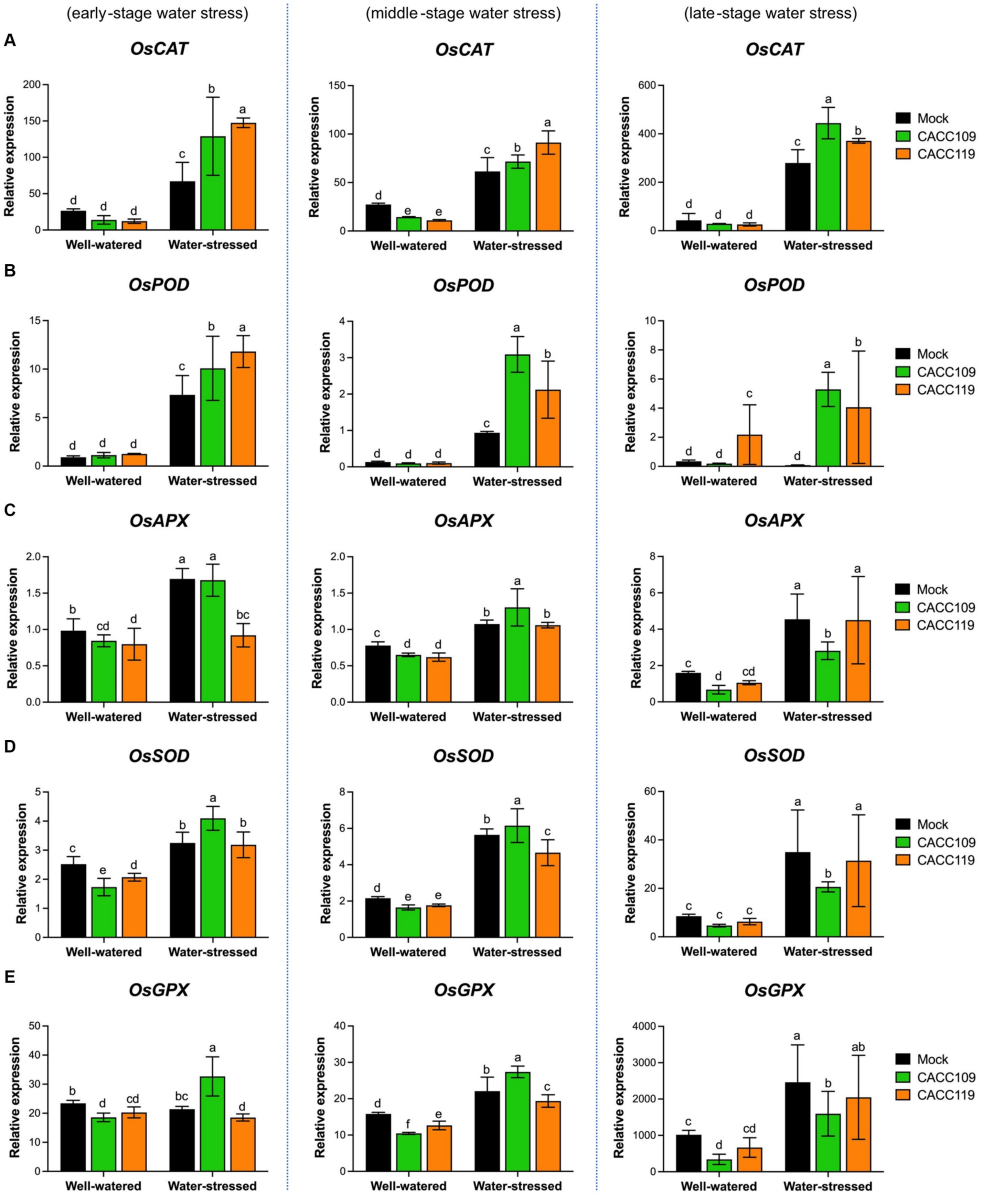
Effects of CACC109 and CACC119 on the expression of antioxidant-related genes in rice leaves. Expression of **(A)** *OsCAT* (*catalase*), **(B)** *OsPOD* (*peroxidase*), **(C)** *OsAPX* (*ascorbate peroxidase*), **(D)** *OsSOD* (*superoxide dismutase*), and **(E)** *OsGPX* (*glutathione peroxidase*) in mock-, CACC109-, and CACC119-treated plants grown under well-watered or water-stressed conditions. Six-week-old plants were subjected to drought stress by maintaining soil water potential at 20–40% for 37 days. Leaf tissue was collected from three independent plants treated with mock, CACC109, or CACC119 in each of the two pots under well-watered and water-stressed conditions at 10 (early), 20 (middle), and 35 (late) days after drought treatment. Data were normalized by the comparative cycle threshold method with *OsActin* as the internal control and presented as relative expression. Values are mean ± SD of two independent experiments. Different letters indicate significant differences (*p* < 0.05, two-way ANOVA and Tukey’s test).

We analyzed the time-course expression of antioxidant-related genes, such as *catalase* (*CAT*), *superoxide dismutase* (*SOD*), *peroxidase* (*POD*), *ascorbate peroxidase* (*APX*), and *glutathione peroxidase* (*GPX*), in the mock-, CACC109-, and CACC119-treated plants under well-watered and water-stressed conditions. Under non-stressed conditions, no significant differences in the expression of antioxidant-related genes were found among the treated plants (Figure 6). However, under water stress, the expression levels of these genes were significantly higher in the mock-, CACC109-, and CACC119-treated plants than in their respective non-stressed controls (Figure 6). Specifically, during drought, the levels of *OsCAT* and *OsPOD* were significantly higher in the CACC109- and CACC119-treated plants than in the mock-treated controls (Figures 6A,B). At the early stage of water stress, *OsAPX* expression was higher in the mock- and CACC109-treated plants than in the CACC119-treated plants. As the stress continued, *OsAPX* expression became higher in the CACC109-treated plants than in the mock- and CACC119-treated plants. However, in the late stages of stress, *OsAPX* expression was notably higher in the mock- and CACC119-treated plants than in the CACC109-treated plants (Figure 6C). The expression levels of *OsSOD* and *OsGPX* were higher in the CACC109-treated plants than in the mock- and CACC119-treated plants at the early and middle stages but lower at the late stage of drought (Figures 6D,E).

Antioxidant activity was significantly higher in the PGPR-treated plants under water stress than in those under control conditions. Additionally, the expression of antioxidant-related genes was modulated below a certain threshold because of the enhanced stress tolerance mediated by the PGPR.

### 3.7 Effects of CACC109 and CACC119 on the expression of DRGs

**Figure 7:**
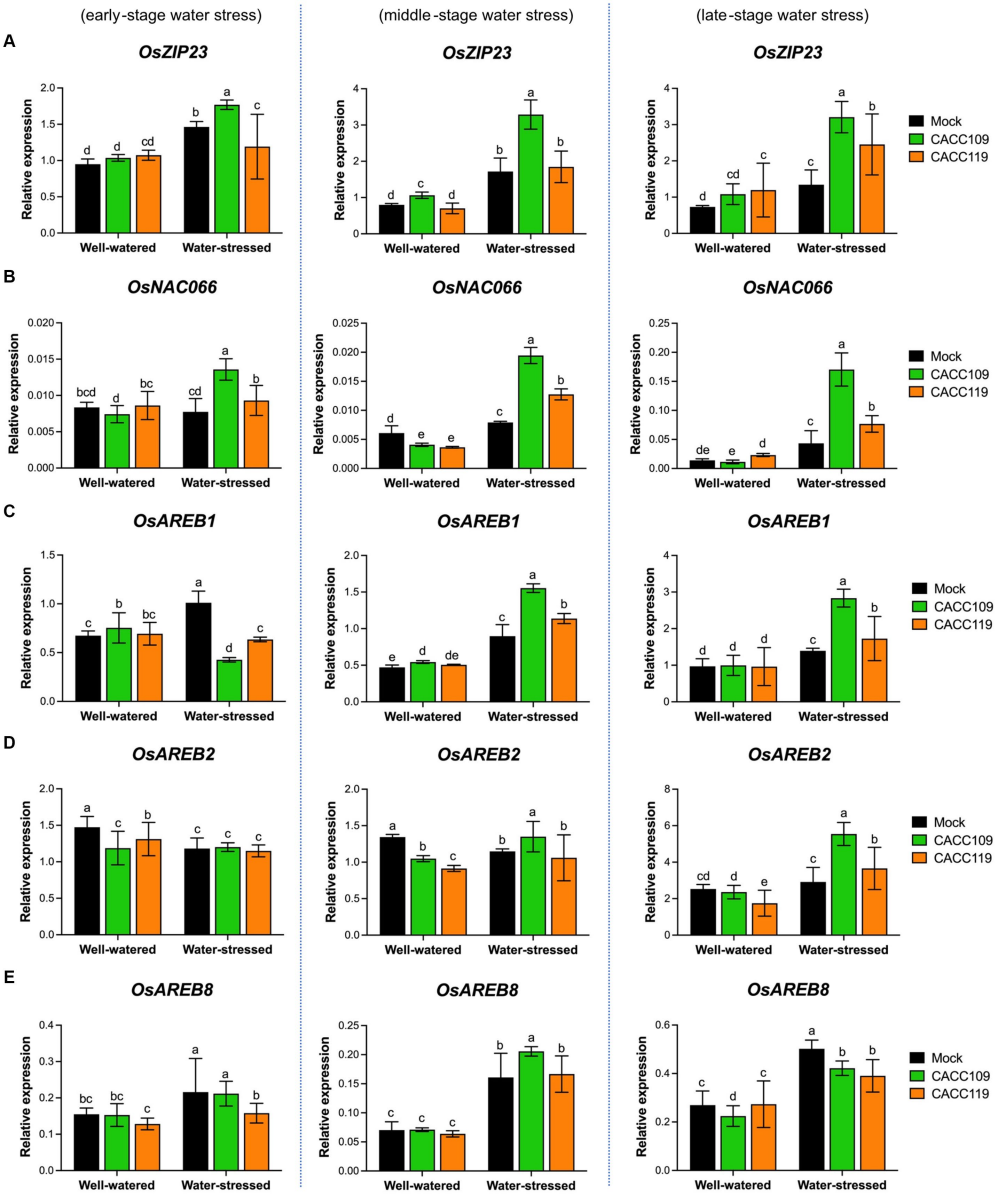
Effects of CACC109 and CACC119 on the expression of ABA-dependent drought-responsive genes in rice leaves. Expression of **(A)** *OsZIP23*, **(B)** *OsNAC066*, **(C)** *OsAREB1*, **(D)** *OsAREB2*, and **(E)** *OsAREB8* in mock-, CACC109-, and CACC119-treated plants grown under well-watered or water-stressed conditions. Six-week-old plants were subjected to drought stress by maintaining soil water potential at 20–40% for 37 days. Leaf tissues were collected from three independent plants treated with mock, CACC109, or CACC119 in each of the two pots under well-watered and water-stressed conditions at 10 (early), 20 (middle), and 35 (late) days after drought treatment. The expression levels of drought-responsive genes were estimated by quantitative real-time PCR, and *OsActin* was used as an internal control for normalization. Values are mean ± SD of two independent experiments. Different letters indicate significant differences (*p* < 0.05, two-way ANOVA and Tukey’s test).

**Figure 8:**
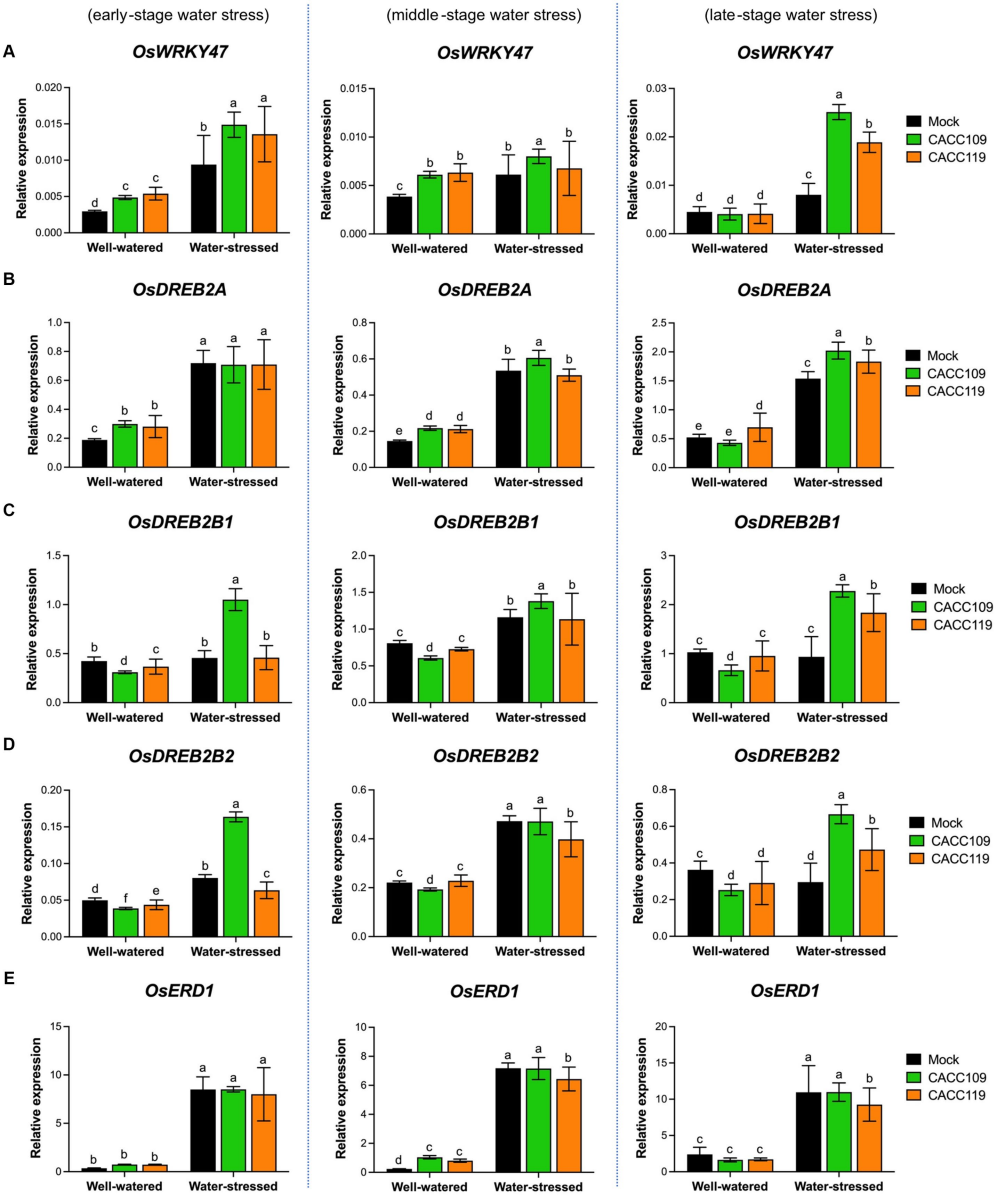
Effects of CACC109 and CACC119 on the expression of ABA-independent drought-responsive genes in rice leaves. Expression of **(A)** *OsWRKY47*, **(B)** *OsDREB2A*, **(C)** *OsDREB2B1*, **(D)** *OsDREB2B2*, and **(E)** *OsERD1* in mock-, CACC109-, and CACC119-treated plants grown under well-watered or water-stressed conditions. Six-week-old plants were subjected to drought by maintaining soil water potential at 20–40% for 37 days. Leaf tissues were collected from three independent plants treated with mock, CACC109, or CACC119 in each of the two pots under well-watered and water-stressed conditions at 10 (early), 20 (middle), and 35 (late) days after drought treatment. Expression levels of drought-responsive genes were estimated by quantitative real-time PCR, and *OsActin* was used as an internal control for normalization. Values are mean ± SD of two independent experiments. Different letters indicate significant differences (*p* < 0.05, two-way ANOVA and Tukey’s test).

The above results suggest that CACC109 and CACC119 positively affect plant responses to drought. Drought triggers a significant reprogramming of gene expression (Shinozaki and Yamaguchi-Shinozaki, 2006). To gain insights into the molecular mechanisms underlying PGPR-induced drought tolerance, we analyzed the expression of ABA-dependent [e.g., *OsbZIP23* (basic region/leucine zipper motif), *OsNAC066* (NAM, ATAF1/2, and CUC2), *OsAREBs* (ABA-responsive element binding protein)] and ABA-independent DRGs [e.g., *OsWRKY47*, *OsDREB2s* (dehydration-responsive element binding protein), and *OsERD1* (early response to dehydration1)] in the mock-, CACC109-, and CACC119-treated plants with or without water stress. Under well-watered conditions, no significant differences in the expression of all DRGs were found between the leaf tissues from the mock- and PGPR-treated plants (Figures 7, 8). The expression levels of all DRGs were significantly higher in the mock-, CACC109-, and CACC119-treated plants under water stress than in their well-watered counterparts, indicating that all plants were affected by drought (Figures 7, 8).

Analysis of the expression of ABA-dependent DRGs showed that during water stress, *OsZIP23* expression peaked in the CACC109-treated plants, whereas the CACC119-treated plants exhibited notably higher *OsZIP23* expression than the mock-treated plants at the late stage (Figure 7A). Meanwhile, *OsNAC066* expression was highest in the CACC109-treated plants, followed by the CACC119-treated plants, and then the control plants at all time points after drought (Figure 7B). At the early stage of water stress, the expression levels of *OsAREB1* and *OsAREB2* were similar or lower in the PGPR-treated plants than in the mock-treated plants. However, as the stress progressed to the middle and late stages, the expression of these genes became significantly higher in the CACC109- and CACC119-treated plants than in the mock-treated plants (Figures 7C,D). The expression of *OsAREB8* showed no noticeable difference between the mock- and PGPR-treated plants under water stress (Figure 7E).

Analysis of the expression of ABA-independent DRGs displayed that at the early stage of water stress, the CACC109- and CACC119-treated plants showed significantly higher *OsWRKY47* expression than the mock-treated plants. However, at the middle stage of water stress, all plants exhibited similar expression levels of *OsWRKY47*. At the late stage, *OsWRKY47* expression was significantly upregulated in the PGPR-treated plants compared with the mock-treated plants (Figure 8A). At the early and middle stages of water stress, the expression of *OsDREB2A* was similar in both the PGPR-treated and mock-treated plants. However, in the late stage, the CACC109- and CACC119-treated plants showed significantly higher levels of *OsDREB2A* expression compared to the mock-treated plants (Figure 8B). The expression levels of both splice variants of *OsDREB2B*, *OsDREB2B1* and *OsDREB2B2*, were higher in the CACC109-treated plants than in the CACC119- and mock-treated plants at the early stage of water stress. At the middle stage, the PGPR- and mock-treated plants exhibited comparable expression levels of *OsDREB2B1* and *OsDREB2B2*. However, at the late stage, the expression levels of *OsDREB2B1* and *OsDREB2B2* were significantly higher in the PGPR-treated plants than in the mock-treated plants, which displayed similar levels to the non-stressed plants (Figures 8C,D). No significant difference in *OsERD1* expression was found between the mock- and PGPR-treated plants under water stress (Figure 8E).

These results indicated that CACC109 and CACC119 enhanced the drought tolerance in rice plants by upregulating the expression of DRGs via ABA-dependent and -independent pathways.

### 3.8 Genome analysis of CACC109 (PRJNA932602) and CACC119 (PRJNA1049983)

Whole-genome sequencing of *B. megaterium* CACC109 and CACC119 yielded 672 and 621 Mb of data with approximately 34× and 5× coverage, respectively. Sequencing reads with a Phred score greater than Q30 were used for *de novo* assembly using PacBio sequencing data. The Microbial Assembly resulted in draft genomes of ~5.3 and 5.7 Mb (5,309,578 and 5,703,136 bp) with 4 and 10 contigs with N_50_ of 5,083,237 and 4,735,530 bp, respectively. The average GC contents of the CACC109 and CACC119 genomes were 38.1 and 37.8%, respectively. Annotation of the CACC109 and CACC119 draft genomes resulted in 5,423 and 5,787 CDSs with median lengths of 714 and 702 bp, 42 and 49 rRNAs, and 130 and 187 tRNAs, respectively, as predicted by Prodigal 2.6.2 (Supplementary Table S3). These genomic features were visualized in circular maps of the *B. megaterium* CACC109 and CACC119 genomes (Supplementary Figure S2).

We deciphered genes enhancing plant growth and abiotic stress resilience from the entire genomes of CACC109 and CACC119. The presence of genes associated with phosphate solubilization, nitrogen fixation, and IAA production highlighted their potential as PGPs. In addition, the detection of genes related to siderophore production and ACC deaminase activity underscores their ability to thrive under adverse environmental conditions and improve plant tolerance to abiotic stresses (Supplementary Table S4). The identification of genes associated with motility and chemotaxis further indicated that CACC109 and CACC119 are plant-associated bacteria capable of navigating to plant roots in response to plant-secreted substances and nutrients (Supplementary Table S5).

### 3.9 Effects of PGPR on soluble organic components in soil

To investigate whether the PGPR affect the balance of soluble organic components in soil, we compared the levels of P_2_O_5_, Ca^2+^, and K^+^ in soils treated with mock, CACC109, or CACC119. Under water stress, P_2_O_5_ content was significantly higher in the CACC109- and CACC119-treated soils than in the mock-treated soils. Furthermore, the application of both PGPR significantly increased the K^+^ and Ca^2+^ contents in the soils by 22.9 and 31.4%, respectively, compared with the mock treatment (Supplementary Figure S3). These results indicated that the PGPR CACC109 and CACC119 played a role in altering the balance of soluble organic components in soils under drought.

## 4 Discussion

Drought is an abiotic stress that can severely affect the growth and production of crops, including rice, and the agricultural demand for food security (Zhang et al., 2018). Many strategies have been developed to increase the resilience of plants to abiotic stresses, allowing them to thrive in challenging environments (Ashry et al., 2022). Among these, inoculation with PGPR is effective in increasing crop productivity in drought-stressed environments (Rodríguez-Salazar et al., 2009). The enhancement of plant growth by PGPR can be attributed to several mechanisms, including the synthesis of PGP hormones and other PGP activities (Glick, 1995). Over the past two decades, various soil microorganisms have been used to alleviate abiotic stresses in plants. PGPR mitigate the effects of drought on crops (Niu et al., 2018; Jochum et al., 2019; Batool et al., 2020). However, the selection of bacteria with abiotic stress tolerance and *in vitro* PGP properties is crucial to alleviate abiotic stress in plants (Jinal et al., 2021).

In the present study, 31 isolates were obtained from ginseng rhizosphere and field soils, of which 28 were identified as *Bacillus* spp. Among the isolates, *B. megaterium* CACC109 and CACC119 showed comparatively better osmotic stress tolerance (Figure 1A). Both strains exhibited EPS and siderophore production (Pallai, 2005; Naseem et al., 2018), nitrogen fixation (Cattelan et al., 1999), IAA synthesis (Loper and Schroth, 1986), and ACC deaminase activity (Glick, 2014) (Figures 1C–H). Notably, CACC109 showed additional advantages, including higher ACC deaminase activity, phosphate solubilization, and EPS production under osmotic stress, enhancing its ability to promote plant growth and alleviate abiotic stress. Phosphate-solubilizing microorganisms act as biofertilizers (Sivasakthi et al., 2013) that increase phosphorus availability and crop yield (Shi et al., 2014). Nitrogen-fixing *Bacillus* species are promising in agriculture because they facilitate plant nutrient uptake (Novo et al., 2018). The IAA produced by PGPR promotes plant growth and nutrient absorption (Loper and Schroth, 1986; Venturi and Keel, 2016; Gowtham et al., 2017). Microbial EPS production under stress conditions enhances bacterial survival, indicating drought tolerance (Sandhya et al., 2009). Specifically, PGPR produce EPS composed of complex organic macromolecules, including polysaccharides, proteins, and uronic acid. These compounds protect microbial cells by stabilizing their membrane structure against adverse environmental conditions such as drought, high salinity and extreme temperatures (Ojuederie and Babalola, 2017). Siderophores are critical for microbial survival and facilitate iron binding and uptake, particularly under conditions of low iron availability. Higher siderophore levels correlate with improved drought tolerance in plants (Andrews et al., 2003; Arzanesh et al., 2011). ACC deaminase activity in many PGPR helps reduce ET levels in plants, mitigating growth inhibition under stress and thereby promoting plant growth (Glick, 2014).

In the present study, we evaluated the effects of the selected PGPR strains, CACC109 and CACC119, on seed germination, seedling growth, and drought stress in rice plants. Both strains improved rice seed germination and root development under osmotic stress induced by PEG 6000 and mannitol (Figure 2) possibly by enhancing EPS production, which mitigated damage and promoted metabolic processes in the seeds (Bakhshandeh et al., 2020). Similarly, a previous study reported a 50% increase in germination rate under stress conditions with the application of EPS-producing bacterial strains (Tewari and Arora, 2014). CACC109 and CACC119 also significantly improved the drought tolerance of rice plants (Figure 4). Notably, CACC109 showed superior EPS production to CACC119 under high osmotic stress (Figure 1C), which is consistent with the observed phenotype. Bacteria can survive under stressful conditions by producing EPS, which increases water retention, regulates the diffusion of organic carbon sources, and allows irreversible attachment and colonization of roots by forming a network of fibrillar material that binds bacteria to the root surface (Chenu and Roberson, 1996; Bashan and Holguin, 1997). EPS are hydrated compounds containing 97% water in the matrix, protecting both bacteria and plants from desiccation. Plants treated with EPS-producing bacteria exhibit enhanced water retention and drought stress tolerance (Bensalim et al., 1998). Bacterial EPS can form a biofilm on the root surface, ultimately enhancing soil structure and supporting plant development while mitigating the impact of drought stress (Sandhya et al., 2009). EPS produced by various bacterial strains enhance soil permeability by improving soil aggregation and maintaining a higher water potential and better nutrient uptake around roots, resulting in increased plant growth and drought tolerance (Upadhyay et al., 2012; Vejan et al., 2016). Niu et al. (2018) reported that several PGPR strains, including *Enterobacter hormaechei*, *Pseudomonas fluorescens*, and *Pseudomonas migulae*, produce EPS and enhance seed germination and seedling growth under drought conditions. The inoculation of sunflower rhizosphere with the EPS-producing rhizobial strain YAS34 under drought conditions significantly increases the ratio of root-adhering soil per root tissue (Alami et al., 2000). Vurukonda et al. (2016) found that inoculating maize seeds with EPS-producing bacterial strains *Alcaligenes faecalis*, *Pseudomonas aeruginosa*, and *Proteus penneri* increases soil moisture and relative water contents, shoot and root length, leaf area, and plant biomass under drought conditions. The results of present study suggest that EPS-producing CACC109 and CACC119 are promising candidates for enhancing plant stress tolerance and promoting seedling establishment under adverse environmental conditions.

The CACC109- and CACC119-treated rice plants had significantly higher shoot and root lengths, leaf widths, plant weights, and chlorophyll contents than the control plants (Figures 3, 4). Notably, CACC109 treatment substantially increased root biomass due to axial and lateral root development, whereas CACC119 exerted a similar but less pronounced effect (Supplementary Figure S1). This result is consistent with the growth-promoting effects of PGPR reported in previous studies. For example, *Bacillus aryabhattai* AB211 from maize (Bhattacharyya et al., 2017), *Bacillus aryabhattai* SRB02 from soybean roots (Park et al., 2017), *Bacillus* sp. BCLRB2 in saline soil (Hadj Brahim et al., 2019), *Bacillus subtilis* EA-CB0575 from banana roots (Franco-Sierra et al., 2020), and *Bacillus* sp. D5 from saffron corms (Magotra et al., 2021) significantly improve plant growth through various mechanisms, such as synthesis of hormones, facilitation of phosphate uptake, and production of beneficial compounds. Our results highlight the potential benefits of nutrient uptake and enhanced adaptability to adverse abiotic stress conditions in rice plants.

Drought decreases plant growth as essential nutrients and solutes are redirected toward stress-related processes (Brestic et al., 2018). Nonetheless, bacterial inoculation enhances plant growth, resulting in significant increases in root and shoot lengths and leaf area (Lin et al., 2020). Different PGPR strains enhance the growth of various plants under abiotic stresses, such as drought and salinity (Kumari et al., 2016; Creus et al., 2020; Mishra et al., 2020; Muhammad Usman Aslam et al., 2020). In the present study, the application of CACC109 and CACC119 significantly improved the shoot length, leaf width, plant weight, chlorophyll content, and proline production in the rice plants under drought, ultimately increasing the drought tolerance in the PGPR-treated plants compared with the mock-treated plants (Figures 3, 4).

Drought can affect the chlorophyll levels in plant leaves and consequently decrease photosynthesis (Liu et al., 2019; Khayatnezhad and Gholamin, 2021). It can significantly reduce photosynthetic efficiency by altering the leaf area, affecting chlorophyll synthesis, increasing lipid peroxidation, and promoting chlorophyll degradation (Khayatnezhad and Gholamin, 2021). Consequently, plants capable of maintaining higher chlorophyll levels under drought stress may exhibit greater tolerance to water shortage and sustain increased growth and biomass production. This hypothesis is supported by the higher total chlorophyll contents and water retention in the CACC109- and CACC119-treated plants than in the mock-treated plants under drought.

The accumulation of osmolytes such as proline, sugars, and proteins in plants is a key indicator of drought tolerance because it facilitates osmotic adjustment and prevents water loss (Vaishnav and Choudhary, 2019; Karimzadeh et al., 2021). In the present study, proline production and proline biosynthesis gene expression significantly increased in the plants treated with CACC109 and CACC119 under drought (Figure 5). One of the defense strategies of PGPR is to improve the tolerance of plants to drought stress, indicating the substantial contribution of CACC109 and CACC119 in alleviating drought in plants by enhancing their defense mechanisms.

Endogenous signaling molecules such as ROS play crucial roles in plant growth, development, and stress adaptation, acting as double-edged swords because of their dual nature as signaling molecules and potential toxins, especially under drought (Miller et al., 2010; Steffens, 2014). Although excessive ROS accumulation can be detrimental to plant cells, plants can enhance drought tolerance by modulating ROS levels through the activities of various antioxidant enzymes, such as SOD, CAT, POD, and GPX (Blokhina, 2003; Miller et al., 2010; Fang et al., 2015; Nadarajah, 2020). Elevated expression of ROS detoxification genes improves plant drought tolerance by mitigating the deleterious effects of oxidative stress on plant metabolism (Ning et al., 2010). In the present study, the CACC109-treated plants had significantly higher expression of antioxidant-related genes *OsSOD*, *OsAPX*, *OsCAT*, *OsPOD*, and *OsGPX* under drought, whereas the CACC119-treated plants had notably higher expression of *OsCAT* and *OsPOD* compared with the mock-treated plants (Figure 6). These findings suggest that CACC109 and CACC119 influence cellular ROS homeostasis and potentially improve drought tolerance in rice plants by regulating oxidative stress levels. In particular, the relative expression trend of these genes varies over time. Specifically, the relative expression of *OsCAT* and *OsPOD* was higher at the late stage of water stress, whereas that of *OsAPX*, *OsSOD*, and *OsGPX* was lower at the same stage in the plants inoculated with CACC109 (Figure 6). This differential expression of antioxidant genes can be attributed to several factors. The enzymes APX, GPX, CAT, and POD utilize different mechanisms to scavenge hydrogen peroxide (H_2_O_2_) and manage oxidative stress in plants. Ascorbate peroxidase requires an ascorbate and glutathione (GSH) regeneration system, known as the ascorbate-glutathione cycle. In the first reaction of this cycle, catalyzed by APX, H_2_O_2_ and ascorbate produce H_2_O and monodehydroascorbate (Mittler, 2002). Similarly, GPX reduces H_2_O_2_ to H_2_O but uses GSH directly as the reducing agent (Noctor et al., 2014). By contrast, CAT directly converts H_2_O_2_ to H_2_O and 1/2 O_2_ and is primarily involved in detoxifying H_2_O_2_ rather than regulating it as a signaling molecule in plants (Apel and Hirt, 2004). Meanwhile, peroxidase can utilize various substrates to reduce H_2_O_2_, often employing phenolic compounds as electron donors, and is involved in a wide range of physiological processes (Gill and Tuteja, 2010). Changes in the balance of these scavenging enzymes trigger compensatory mechanisms. For instance, a decrease in CAT activity upregulates APX and GPX to maintain ROS homeostasis. Similarly, POD activity may increase in response to stress conditions to compensate for changes in the levels or activity of other antioxidant enzymes, thus contributing to the overall ROS detoxification and stress adaptation (Apel and Hirt, 2004). Inoculation with CACC109 could influence the expression of ROS detoxification genes by modulating plant hormone balance, signaling pathways, or enhancing drought stress-specific response mechanisms. This phenomenon could result in a more targeted activation of *OsCAT* and *OsPOD* while downregulating others such as *OsAPX*, *OsSOD*, and *OsGPX* in the late stage. These observed differential expressions likely reflect the strategic and dynamic response of plants to effectively manage ROS levels. Further studies involving detailed temporal gene expression analysis and the role of CACC109 inoculation may provide deeper insights into these regulatory mechanisms.

Plants have evolved numerous defense mechanisms against drought, among which transcription factors (TFs) play a crucial role in regulating the expression of stress-related genes. Members of the basic leucine zipper (bZIP) family are important TFs responsible for ABA signaling and plant responses to drought (Joo et al., 2021). *Os*bZIP23 is a key regulator of drought and salinity tolerance in rice (Xiang et al., 2008). AREBs, which belong to the group A subfamily of bZIP TFs, play key roles in the ABA signaling pathway in rice. They regulate the expression of stress-responsive genes, especially under drought and salinity, thus contributing to the stress tolerance mechanisms in rice (Yoshida et al., 2010). NAC, one of the largest families of TFs in plants, is involved in the regulation of growth and stress responses (Nakashima et al., 2012; Shao et al., 2015). *OsNAC066* positively regulates oxidative and drought stress tolerance, as demonstrated by functional analyses of overexpression and RNAi transgenic rice plants (Yuan et al., 2019). WRKY TFs play an important role in regulating transcriptional reprogramming associated with tolerance to multiple abiotic stresses in plants (Chen et al., 2012). *OsWRKY47* is a positive regulator of drought response, and *OsWRKY47*-overexpressing rice plants exhibit greater drought tolerance than wild-type plants (Raineri et al., 2015). DREB TFs play critical roles in improving abiotic stress tolerance, mostly in an ABA-independent manner (Dubouzet et al., 2003; Lata and Prasad, 2011). In rice, *OsDREB2A* expression is induced by water stress, resulting in increased drought tolerance (Cui et al., 2011). The *OsDREB2B* transcript exhibits functional and non-functional forms under drought, with alternative splicing induced by its pre-mRNA, thereby enhancing drought tolerance (Matsukura et al., 2010). These findings highlight the critical role of *OsDREB2* in regulating drought tolerance in rice plants.

In the present study, we investigated the molecular mechanisms underlying PGPR-induced drought tolerance by examining the expression of *DRGs*. CACC109 and CACC119 positively influenced the response of rice plants to drought. Under well-watered conditions, the mock- and PGPR-treated plants showed no significant difference in *DRG* expression (Figures 7, 8). However, under water stress, the PGPR- and mock-treated plants showed significantly higher expression of *DRGs* than the control plants. *OsZIP23* expression peaked in the CACC109-treated plants. *OsDREB2A* expression was significantly higher in both CACC-treated plants than in the mock-treated plants at the late drought stage. The expression of *OsDREB2B1* and *OsDREB2B2* peaked in the CACC109-treated plants during water stress. The highest expression levels of *OsNAC066* were observed in the CACC109-treated plants. The expression levels of *OsAREB1* and *OsAREB2* were significantly higher in both CACC-treated plants than in the mock-treated plants at the middle and late stages of water stress. However, *OsAREB8* expression was similar or significantly lower in the CACC-treated plants at these same stages. The *AREB* family members, though functionally related, can have different regulatory controls and promoter regions responding to specific signals. The inoculation with CACC could interact differently with the signaling pathways involving *OsAREB8* compared to *OsAREB1* and *OsAREB2*. This could result in a distinct modulation of *OsAREB8* expression, possibly due to specific hormonal changes or stress signals induced by CACC treatment. Both PGPR-treated plants exhibited significantly higher *OsWRKY47* expression at the early and late stages of water stress. Moreover, the expression of *OsDREB2B1* and *OsDREB2B2* was significantly upregulated in the CACC109-treated plants during the early stage and in both PGPR-treated plants at the late stage of drought. These results suggest that CACC109 and CACC119 enhance drought tolerance in rice via ABA-dependent and -independent pathways, as reflected by the altered expression of the abovementioned DRGs. Taken together, the results of our study highlight the beneficial effects of PGPR CACC109 and CACC119 on plant response to drought and provide novel insights into the molecular mechanisms underlying PGPR-induced drought tolerance in rice.

The whole genome analysis of CACC109 and CACC119 reveals a suite of genes that underscore their potential as effective PGPR, particularly under stress conditions. The genomes of these strains encode genes associated with key physiological traits that enhance plant growth and stress resilience. For instance, the presence of genes related to phosphate solubilization, nitrogen fixation, and IAA production highlights their ability to improve nutrient availability and hormonal balance, which are crucial for plant growth and stress adaptation (Supplementary Table S4) (Lugtenberg and Kamilova, 2009). Moreover, the detection of genes involved in siderophore production and ACC deaminase activity points to their role in mitigating the adverse effects of abiotic stresses. Siderophores can help plants acquire iron under limited conditions, while ACC deaminase can reduce the levels of ET, a hormone that often exacerbates stress responses (Hayat et al., 2010; Singh et al., 2022). The presence of genes related to motility and chemotaxis further indicates that these bacteria can navigate toward plant roots, enhancing their colonization and interaction with plant roots in response to plant-secreted signals and nutrient gradients (Supplementary Table S5) (Feng et al., 2021). These genomic insights are instrumental in understanding how CACC109 and CACC119 can positively impact rice growth under drought stress. The array of beneficial traits encoded in their genomes supports their potential as effective PGPR, capable of not only promoting growth but also enhancing stress resilience. Future research should focus on validating these genomic predictions in practical field settings, particularly under varied stress conditions, to fully elucidate their roles and optimize their application for crop improvement.

The PGPR have long been recognized for their beneficial effects on plant growth under optimal conditions. However, their efficacy can be complex when plants face different types of stress. Recent studies suggest that while PGPR can significantly enhance plant growth, their benefits might be context-dependent, especially under stress conditions. For instance, under deficit stresses such as drought, the growth-promoting effects of PGPR might be counterproductive as they could divert the plant’s energy away from crucial stress responses, leading to potential negative outcomes (Glick, 2012; Vurukonda et al., 2016). Conversely, under cumulative stresses, such as salinity, PGPR might help alleviate some of the stress impacts through mechanisms such as dilution effects, which can help reduce the severity of stress impacts on plant growth (Kumar et al., 2023). In the present study, the strains CACC109 and CACC119 demonstrated positive effects on rice plants under drought stress (Figures 3, 4). This finding is consistent with the notion that some PGPR strains can enhance plant resilience to specific stress conditions. However, the response of these strains to cumulative stresses, including combinations of drought and salinity, remains unexplored. Further research is warranted to understand how these PGPR strains perform under such combined stresses. This would provide a comprehensive view of their functional capabilities and help differentiate between mere growth promotion and genuine alleviation of stress-induced growth inhibition, offering deeper insights into the interplay between PGPR and environmental stressors.

While this study offers valuable insights into the potential of CACC109 and CACC119 as PGPR for promoting plant growth and enhancing drought tolerance in rice plants, the limitations of the present study should be acknowledged. Firstly, the study focused only on rice plants, and the efficacy of these PGPR strains may vary across different plant species or even across cultivars of the same species. Additionally, the experimental conditions may not accurately replicate real agricultural environments, potentially affecting the generalizability of the findings. Moreover, the mechanisms underlying the observed effects of the PGPR strains have not been fully elucidated, indicating the need for further research to comprehensively understand their mode of action. Furthermore, the long-term effects of PGPR inoculation on soil health and ecosystem dynamics were not investigated in this study, underscoring the necessity for future studies to assess the broader ecological implications of PGPR use in agriculture.

## 5 Conclusion

This study aimed to identify novel PGPR strains that can promote plant growth and alleviate drought stress. *B. megaterium* strains CACC109 and CACC119 displayed various PGP activities. They significantly enhanced rice seed germination under osmotic stress and promoted root growth under stressed and non-stressed conditions. Additionally, these strains improved overall plant growth parameters, such as shoot and root lengths and weights, and leaf widths, while also enhancing plant physiological characteristics, such as chlorophyll levels and osmolyte production. Notably, the rice plants treated with CACC109 and CACC119 exhibited enhanced drought tolerance, as evidenced by their higher survival rates, increased chlorophyll and proline contents, reduced water loss rates, and upregulated expression of antioxidant-related and drought-responsive genes. Overall, this study provides valuable insights into the beneficial effects of PGPR on plant growth and their response to environmental stressors, offering promising avenues for increasing crop productivity and mitigating the impacts of climate change on agriculture.

## Data Availability

The datasets presented in this study can be found in online repositories. The names of the repository/repositories and accession number(s) can be found in the article/Supplementary material.

## Footnotes

^1^ http://www.ncbi.nlm.nih.gov

^2^ http://eggnogdb.embl.de

## Supplementary material

SUPPLEMENTARY FIGURE S1 Effects of CACC109 and CACC119 on rice shoot growth and root development. **(A)** Comparison of shoot growth and root development in 9-week-old mock-, CACC109-, and CACC119-treated rice plants grown under well-watered conditions. **(B)** Magnification of the root parts.

SUPPLEMENTARY FIGURE S2 Circular map of the genome and genome features circles of *B. megaterium* CACC109 **(A)** and CACC119 **(B)**. From the outside to the inside, the outer black circle represents the scale line in Mbps; the second gray band represents contigs; the third and fourth circles represent the annotated reference genes (specifically, CDSs) found in the forward and reverse strands, respectively; the fifth circle displays only rRNA and tRNA found in the genome; the sixth circle displays the GC skew metric. The genomic mean GC-skew value is used as the baseline, relative to which higher-than-average values are displayed in green, whereas lower-than-average values are displayed in red; the seventh circle displays the GC ratio metric. GC ratio also uses the genomic mean GC ratio value as its baseline, with higher-than-average values in blue and lower-than-average values in yellow.

SUPPLEMENTARY FIGURE S3 Effects of CACC109 and CACC119 on soluble organic components. Comparison of soil nutrients (P_2_O_5_, K^+^, and Ca^2+^) between mock and PGPR (CACC109 and CACC119)-treated soils in pots. Data are presented as the mean ± SD of at least three independent samples. Different letters indicate significant differences (*P* < 0.05, Student’s *t*-test). The experiment was performed three times.
