# Supplementary figures and images for: Plant growth-promoting rhizobacterium Bacillus megaterium modulates the expression of antioxidant-related and drought-responsive genes to protect rice (Oryza sativa L.) from drought
Source: Front Microbiol. 2024 Aug 21;15:1430546. doi: 10.3389/fmicb.2024.1430546 (PMC11371581; doi:10.3389/fmicb.2024.1430546)

## Supplementary Figure S1

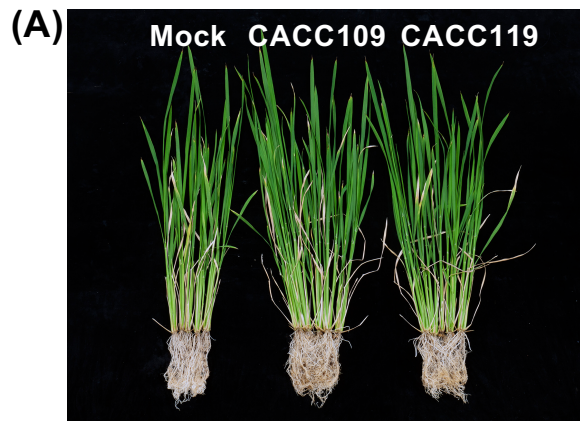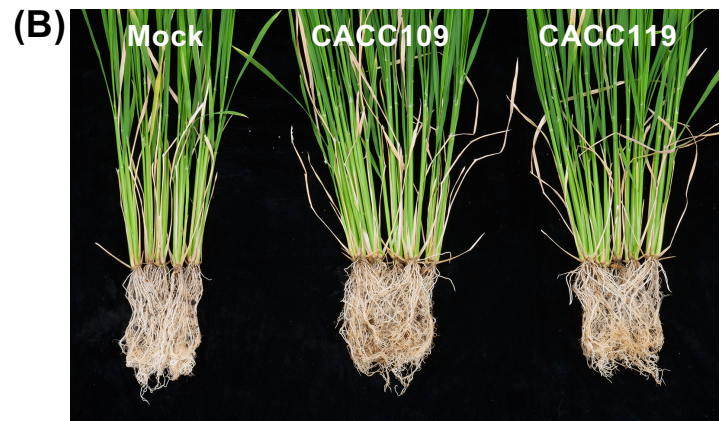

Supplement: SUPPLEMENTARY FIGURE S1 — Effects of CACC109 and CACC119 on rice shoot growth and root development. (A) Comparison of shoot growth and root development in 9-week-old mock-, CACC109-, and CACC119-treated rice plants grown under well-watered conditions. (B) Magnification of the root parts. [file Image_1.pdf]

Supplementary Figure S2

(A)

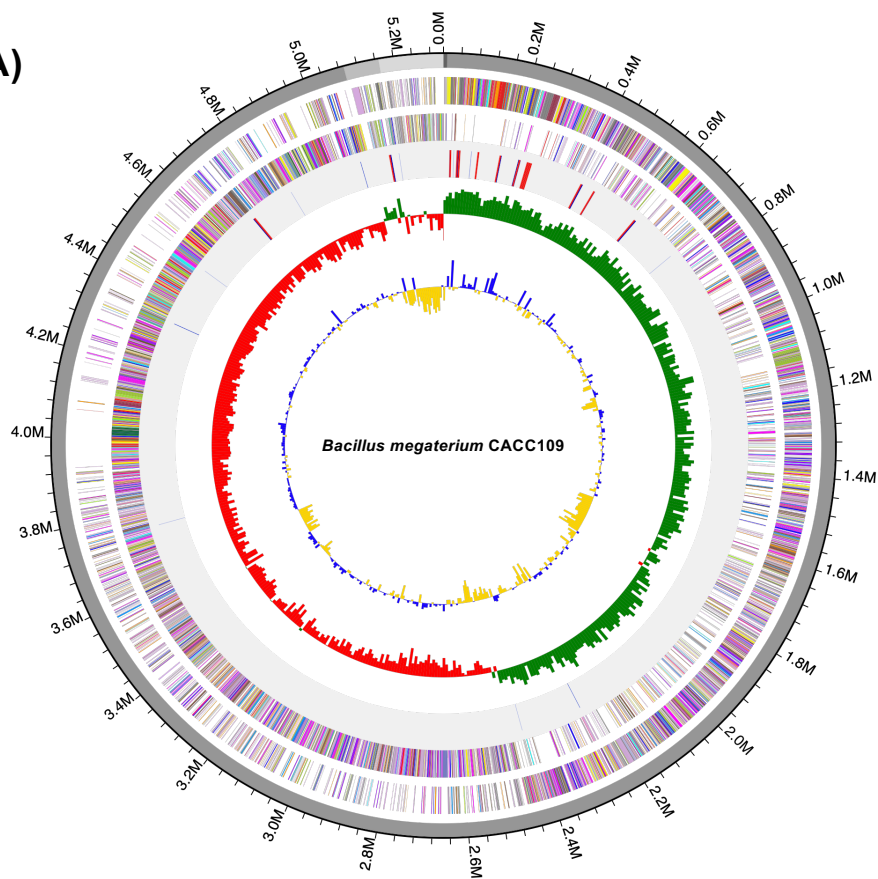

(B)

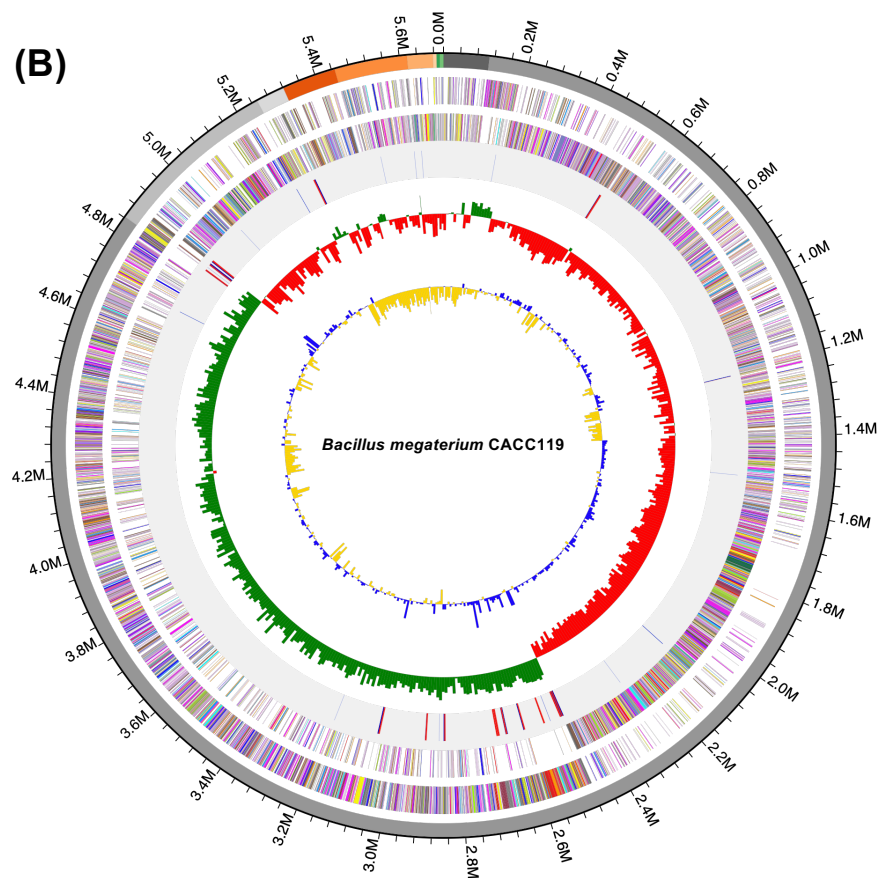

Supplement: SUPPLEMENTARY FIGURE S2 — Circular map of the genome and genome features circles of B. megaterium CACC109 (A) and CACC119 (B). From the outside to the inside, the outer black circle represents the scale line in Mbps; the second gray band represents contigs; the third and fourth circles represent the annotated reference genes (specifically, CDSs) found in the forward and reverse strands, respectively; the fifth circle displays only rRNA and tRNA found in the genome; the sixth circle displays the GC skew metric. The genomic mean GC-skew value is used as the baseline, relative to which higher-than-average values are displayed in green, whereas lower-than-average values are displayed in red; the seventh circle displays the GC ratio metric. GC ratio also uses the genomic mean GC ratio value as its baseline, with higher-than-average values in blue and lower-than-average values in yellow. [file Image_2.pdf]

Supplementary Figure S3

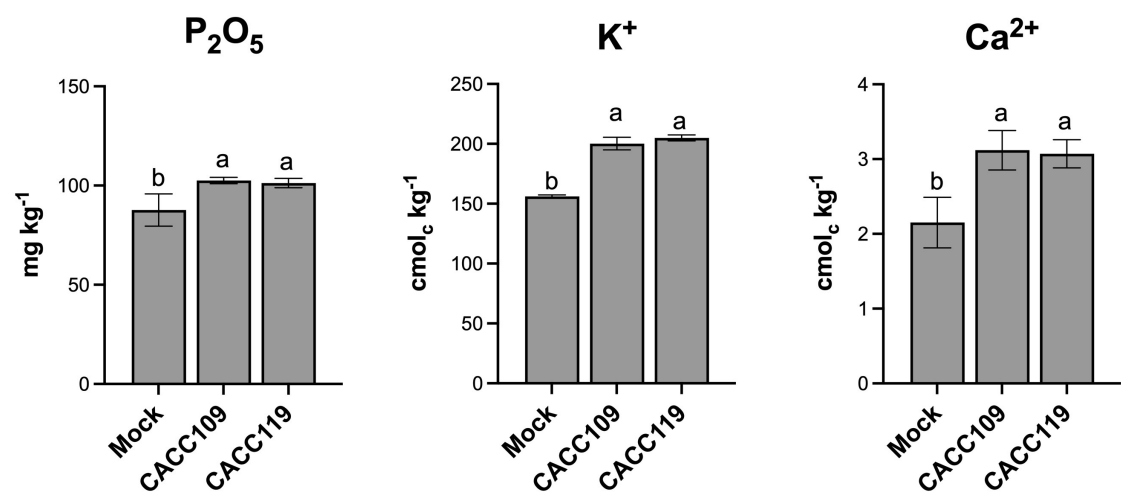

Supplement: SUPPLEMENTARY FIGURE S3 — Effects of CACC109 and CACC119 on soluble organic components. Comparison of soil nutrients (P2O5, K+, and Ca2+) between mock and PGPR (CACC109 and CACC119)-treated soils in pots. Data are presented as the mean ± SD of at least three independent samples. Different letters indicate significant differences (P < 0.05, Student’s t-test). The experiment was performed three times. [file Image_3.pdf]
